# Integrated transcriptome and proteome analyses reveal candidate genes for ginsenoside biosynthesis in *Panax japonicus* C. A. Meyer

**DOI:** 10.3389/fpls.2022.1106145

**Published:** 2023-01-09

**Authors:** Chaokang Huang, Pengfei Li, Xiaolin Yang, Tengfei Niu, Shujuan Zhao, Li Yang, Rufeng Wang, Zhengtao Wang

**Affiliations:** ^1^ The SATCM Key Laboratory for New Resources and Quality Evaluation of Chinese Medicines, Institute of Chinese Materia Medica, Shanghai University of Traditional Chinese Medicine, Shanghai, China; ^2^ The MOE Key Laboratory for Standardization of Chinese Medicines, Institute of Chinese Materia Medica, Shanghai University of Traditional Chinese Medicine, Shanghai, China

**Keywords:** Panx japonicus, ginsenoside, biosynthesis, metabolite profile, transcriptome, proteomics

## Abstract

*Panax japonicus* C. A. Meyer is a plant of the Araliaceae family, and its rhizomes can be used as dietary supplements. It is extremely rich in bioactive components ginsenosides with benefits to human health. However, the underlying mechanisms of ginsenosides biosynthesis in *Panax japonicus* remains poorly understood. Therefore, a comprehensive analysis of the metabolites, transcriptome, and proteome was conducted to investigate ginsenoside metabolism of *Panax japonicus*. Here, three types of ginsenosides were found to exhibited tissue-specific distribution using the liquid chromatography–mass spectrometry method. Next, differentially expressed gene analysis revealed that transcript levels of ginsenosides biosynthetic genes have significant differences between differential samples. In addition, correlation analysis showed that the ginsenosides content was closely related to the expression level of 29 cytochrome P450s and 92 Uridine diphosphate-glycosyltransferases. Finally, phylogenetic analysis was performed for the target proteins to conduct preliminary studies on their functions and classification. This study provides insight into the dynamic changes and biosynthetic pathway of ginsenosides and offers valuable information on the metabolic regulation of *Panax japonicus*.

## Introduction

*Panax japonicus* C. A. Meyer (*P. japonicus*), belonging to the Araliaceae family and *Panax* genus, is a kind of slow-growing perennial medicinal plant. *P. japonicus* commonly grows wild in cooler climates in eastern Asia including China, Japan, and Korea (Rai et al., 2016). The rhizome of *P. japonicus* named “Zhujieshen” (ZJS) has been used as one of traditional Chinese medicines (TCM) and dietary supplement for more than 2,000 years in China. ZJS is also used as a substitute for ginseng root in Chinese ethnic minorities like the Tujia nationality (He et al., 2012). It has been identified in the effects of eliminating stasis to stop pains, suppressing cough, dispelling phlegm, strengthening, and nourishing from the ancient theories and clinical practices of TCM (Chinese Pharmacopeia Commission, 2020).

In recent years, researchers have demonstrated that the extracts from rhizomes of *P. japonicus* had various pharmacological properties, such as anti-ulcer, anti-obesity, hematopoietic effect, anti-fatigue, anti-oxidant, anti-cancer, and immuno-regulation (Han et al., 2005; Yang et al., 2014; Zhang et al., 2014; Zhang et al., 2015a; Harun et al., 2020). In fact, most of the secondary metabolites as natural products in medicinal plants have been verified that responsible for pharmacological activities (Nett et al., 2021). For the medicinal plants of the *Panax* genus, most of the therapeutic effects are derived from triterpenoid saponin generally named ginsenosides (Zhang et al., 2015a). So far, more than 280 different types of ginsenosides have been isolated and identified from the *Panax* genus. Ginsenosides can be roughly divided into two categories by differentiated aglycones: pentacyclic saponins in oleanene-type (OA-type) and tetracyclic saponins in dammarane-type (DA-type) further subdivided into protopanaxadiol-type (PPD-type), protopanaxatriol-type (PPT-type) and ocotillol-type (OCT-type) (Jin et al., 2013). The plants of *Panax* genus were classified into three groups based on chemical profiles, and *P. japonicus* was classified in Group III owing to both high amounts of pentacyclic and tetracyclic saponins (Hou et al., 2021). And different types of ginsenosides appear to have tissue-specific accumulation patterns (Xu et al., 2020).

Natural product biosynthesis covers a series of chemical transformations and an accompanying set of specific enzymes, and their distribution and accumulation are related to the expression levels of related enzymes (Nett et al., 2021). Therefore, we hypothesized that the special accumulation of ginsenoside of *P. japonicus* should be regulated by expression level of terpene synthase and aglycone modifying enzyme but the specific mechanisms are unknown. The biosynthetic pathways of ginsenosides involve two key processes as follows: the basic formation of the triterpenoid skeleton and post-modification (Sawai and Saito, 2011; Tang et al., 2022). It has been elucidated that the formation of the triterpenoid skeleton originated from the mevalonate pathway (MVA) and methylerythritol phosphate pathway (MEP) (Yi et al., 2022). Isopentenyl pyrophosphate (IPP) generated from MVA and MEP pathways was catalyzed by squalene synthases and squalene monooxygenases to synthesize 3(*S*)-2,3-oxidosqualene. 3(*S*)-2,3-oxysqualene was cyclized by oxidosqualene cyclases (OSCs) to generate various triterpenoid skeletons. In the second process, cytochrome P450s (CYP450s) and UDP-glycosyltransferases (UGTs) played very critical roles in the modifications of triterpenoid skeletons to generate ginsenosides. CYP450 is a heme-containing oxygenase that can introduce oxygen atoms in oxygen molecules into hydrophobic substrates to generate more water-soluble products (Urlacher and Girhard, 2012). The oxygen atom introduced by CYP450 provides anchors for further glycosyl modification. Glycosylation catalyzed by UGTs is the most widespread chemical modification leading to structural diversity of ginsenoside. Most plant UGTs belong to GTs superfamily 1 and were assigned to the UGT 71–100 family defined by the presence of a 44 amino acid C-terminal characteristic motif called PSPG (plant secondary product glycosyltransferase)-box (Hughes and Hughes, 1994). Therefore, decoding the genetics of medicinal plants is very significant for understanding their physiological characteristics and tracing interested genes. However, potential genes associated with ginsenosides biosynthesis in *P. japonicus* is still unclear.

With the development of multiple “omics” platforms including genomics, transcriptomics, metabolomics and proteomics, the term “phytochemical genomics” has originated from systematically integrating above “omics” types of researches. This systematic integration drives the discovery of new natural products as well as analysis of natural product biosynthetic pathways (Boopathi et al., 2020). Previous studies have shown that ginsenosides were already synthesized in the first year of growth of ginseng plants, and the contents and diversities of ginsenosides had significant changes in different tissue in the early growth stage (Fang et al., 2022; Luu et al., 2022). To the best of our knowledge, all reported transcriptome sequencing studies for *P. japonicus* have been performed with their mature materials as research objects (Xu et al., 2020). In 2015, the 6-year-old rhizome of *P. japonicus* was sequenced with next-generation short-read sequencing technology (Illumina) to explore triterpenoid biosynthetic pathways (Zhang et al., 2015b). Similarly, Amit Rai et al. performed Illumina sequencing on multiple parts of 7-year-old *P. japonicus* and some specific transcripts in biosynthetic pathways of saponins were investigated (Rai et al., 2016). Although previous efforts have studied the genes for biosynthetic pathways of ginsenosides in *P. japonicus* by transcriptome sequencing, overall understanding of tissue-specific expression patterns of ginsenosides and related biosynthetic mechanism are still quite incomplete.

Transcriptomic-guided differential expression analyses have been proven for discovering plant biosynthetic genes. However, for those plants lacking sequenced genomes, gene expression analysis relied on *de novo* assembly of short reads from Illumina sequencing often results in fragmented or misassemble contigs that mislead the analysis (Nett et al., 2021). Therefore, PacBio single-molecule real-time (SMRT) sequencing were utilized to obtain full-length complementary DNA (cDNA) reads of RNA isolated from *P. japonicus* tissues to generate a reference transcriptome to avoid the technical limitations of *de novo* transcriptome assembly. Illumina sequencing libraries from multiple plant tissues were then used to quantify relative expressions within this full-length transcriptome. It is well known that proteins are the actual cellular effectors, and the in-depth studies on metabolic pathways of ginsenosides are inseparable from the analysis at the protein level (Li et al., 2021). Proteomics is the next step in the study of the central dogma after genomics and transcriptomics that can provide much more rich and accurate information on protein abundance. In recent years, researchers have also paid more attention to proteomic research of *Panax* genus such as *P. notoginseng* and carried out the identification of stress response proteins and enzymes related to ginsenoside biosynthesis (Li et al., 2021). Therefore, combinatorial omics techniques can enable us to better disclose potential genes associated with ginsenosides biosynthesis in *P. japonicus*.

In this work, the dynamic changes of ginsenosides in *P. japonicus* through quantitative analysis were performed by liquid chromatography-mass spectrometry (LC-MS). The molecular mechanism underlying the changes in ginsenosides was further explored at molecular levels *via* a combined analysis of the metabolites, transcriptome, and proteome. This study provides insight into the dynamic changes and biosynthetic pathway of ginsenosides and offers a comprehensive metabolomic, transcriptomic and proteomic overview of *P. japonicus* in early growth stage. Moreover, it provides a valuable basis and reference for *P. japonicus* breeding and *de novo* biosynthesis of rare ginsenosides.

## Materials and methods

### Chemical reagents and plant materials

Ginsenoside standards were purchased from Purifa (Chengdu, China). Formic acid and acetonitrile were obtained from Fisher Scientific (Pittsburgh, PA). Different growth ages of *P. japonicus* in 1yr, 2yr, and 3yr were collected from Enshi Tujia and Miao autonomous prefecture, Hubei, China. The rhizome, leaves, stems, and lateral roots (designated as R, L, S, and LR) of *P. japonicus* were separated, frozen in liquid nitrogen and stored at -80°C for transcriptome and proteome experiments. Each sample was taken in three copies used as a separate sample for RNA-seq and proteomic sequencing.

### Quantitative analysis of ginsenosides in P. japonicus

Ginsenosides were accurately weighed and dissolved in methanol-water (60:40, *v/v*). Then stock solutions were appropriately diluted to various concentrations from 1 ng/mL to 40 *μ*g/mL as working solutions. *P. japonicus* samples were powdered and dried at 55°C. Each sample (10 mg) was accurately weighed and ultrasonically extracted with 1.25 mL of 60% aqueous methanol at 25°C for 40 min. The extract was centrifuged at 16,000 g for 30 min, and the supernatant was filtered through a 0.22 *μ*m filter before analysis.

Quantitative analysis was performed on a Shimadzu LC-30AD HPLC system (Kyoto, Japan) coupled with a Shimadzu LC/MS-8050 triple quadrupole mass spectrometer in negative multiple reaction monitoring (MRM) mode. Chromatographic separation was performed with column ACQUITY UPLC^®^ HSS T3 (2.1×100 mm, 1.8 *μ*m, Waters) maintained at 30°C at a flow rate of 0.2 mL/min with an injection volume of 1 μL. Solvent A (Acetonitrile: Formic acid = 100:0.1, *v/v*) and solvent B (Water: Formic acid = 100:0.1, *v/v*) were set as mobile phases. Elution system was set as follows: 10% A for 0-2 min, 10%-15% A for 2-4min, 15%-20% A for 4-6 min, 20%-25% A for 6-12 min, 25%-30% for 12-15 min, 30%-35% for 15-35 min, 35%-75% for 35-43 min, 75%-90% for 43-48 min, 90% A for 48-56.01 min, 10% A for 56.01-58 min. All MRM transitions for ginsenosides can be found in **Table S1**. Operation parameters of MS experiments were optimized as follows: nebulizing gas flow: 3 L/min, heating gas flow: 10 L/min, interface temperature: 300°C, DL temperature: 250°C, heat block temperature: 400°C, drying gas flow: 10 L/min.

### RNA-seq and analysis

Plant tissues were homogenized under liquid nitrogen *via* mortar/pestle pretreated with RNaseZap RNase Decontamination Solution (Thermo Fisher Scientific). Total RNA was isolated using the Spectrum Plant Total RNA kit (Millipore Sigma). RNA for SMRT sequencing (Pacific Biosciences, PacBio) was isolated from rhizomes, stems, leaves, and lateral roots. RNA for Illumina sequencing was conducted as the same general strategies of pooled samples. The Illumina library was separately prepared by each tissue type with three biological replicates (Grabherr et al., 2011; Bolger et al., 2014). RNA was quantified *via* nanodrop and with the Qubit HS (high sensitivity) RNA Assay kit (Thermo Fisher Scientific), and its quality was assured with analysis using an RNA 6000Nano chip on a 2100 Bioanalyzer (Agilent), Illumina (New England Biolabs). All the transcriptomic data used in this study is available from the NCBI Sequence Read Archive database (SRA; http://www.ncbi.nlm.nih.gov/sra) under accession number PRJNA885251.

### Total protein extraction and analysis

Plant samples were ground to powder in liquid nitrogen and then transformed in SDT buffer (4% SDS, 100 mM Tris-HCl, 1 mM DTT, pH7.6) was used for sample lysis and protein extraction. Proteins were quantified with the BCA Protein Assay Kit (Bio-Rad, USA), and qualitied by SDS polyacrylamide gel electrophoresis, and then the proteins were stored at −80°C for further analysis. Protein digestion by trypsin was performed according to the filter-aided sample preparation (FASP) procedures referred to in previous work (Wiśniewski et al., 2009). The digest peptides of each sample were desalted on C18 Cartridges (Empore™ SPE Cartridges C18 bed I.D. 7 mm, volume 3 mL, Sigma), and concentrated by vacuum centrifugation, and then reconstituted in 40 *µ*L of 0.1% (*v/v*) formic acid. The contents of peptides were determined by measuring at 280 nm with NanoDrop 2000 (Thermo Fisher) using an extinction coefficient of 1.1 for a 0.1% (g/L) solution.

LC-MS/MS analysis was performed on a Q Exactive mass spectrometer that was coupled to Easy nLC (Thermo Fisher Scientific) for 120 min. The peptides were loaded onto a reverse-phase trap column connected to a C18-reversed phase analytical column in buffer A (0.1% formic acid) and separated with a linear gradient of buffer B (84% acetonitrile and 0.1% formic acid) at a flow rate of 300 nL/min controlled by IntelliFlow technology. The mass spectrometer was operated in positive ion mode. MS data were acquired using a data-dependent top10 method dynamically choosing the most abundant precursor ions from the survey scan (300–1800 *m/z*) for higher-energy collisional dissociation (HCD) fragmentation. The automatic gain control (AGC) target was set to 3e^6^, and the maximum injects time was set to 10 ms. The dynamic exclusion duration was set as 40.0 s. Survey scans were acquired at a resolution of 70,000 at *m/z* 200. The resolution for HCD spectra was set to 17,500 at *m/z* 200, and the isolation width was 2 *m/z*. The normalized collision energy was 30 eV and the underfill ratio was defined as 0.1%. The instrument was run with peptide recognition mode enabled. The MS raw data for each sample were combined and searched using the MaxQuant 1.5.3.17 software for identification and quantitation analysis. Data are available *via* ProteomeXchange with identifier PXD038579.

### Gene co-expression network analysis

The WGCNA V1.70-3 R package (Langfelder and Horvath, 2008) was applied to conduct co-expression and module analyses. A signed weighted adjacency matrix was generated from the correlation matrix using a soft power threshold of 6, allowing to approximate the scale-free topology of the resulting network, and topological overlap matrix (TOM) was further created. Significant positive modules (*R*>0.6, *P*<0.05) were selected from transcript analyses of all saponin contents. Candidate unigenes involved in saponin biosynthesis were further screened out based on gene annotations, and then the correlation between transcript expression profiles and saponins contents was further analyzed.

### Phylogenetic analysis

Protein sequences were aligned using the MUSCLE program, and Neighbor-Joining tree was constructed with bootstrap values obtained after 10,000 replications using the MEGAX program. R package ggtree (Yu et al., 2017) was used to visualize and decorate phylogenetic trees.

## Result and discussion

### Quantitative analysis of ginsenosides

**Figure 1:**
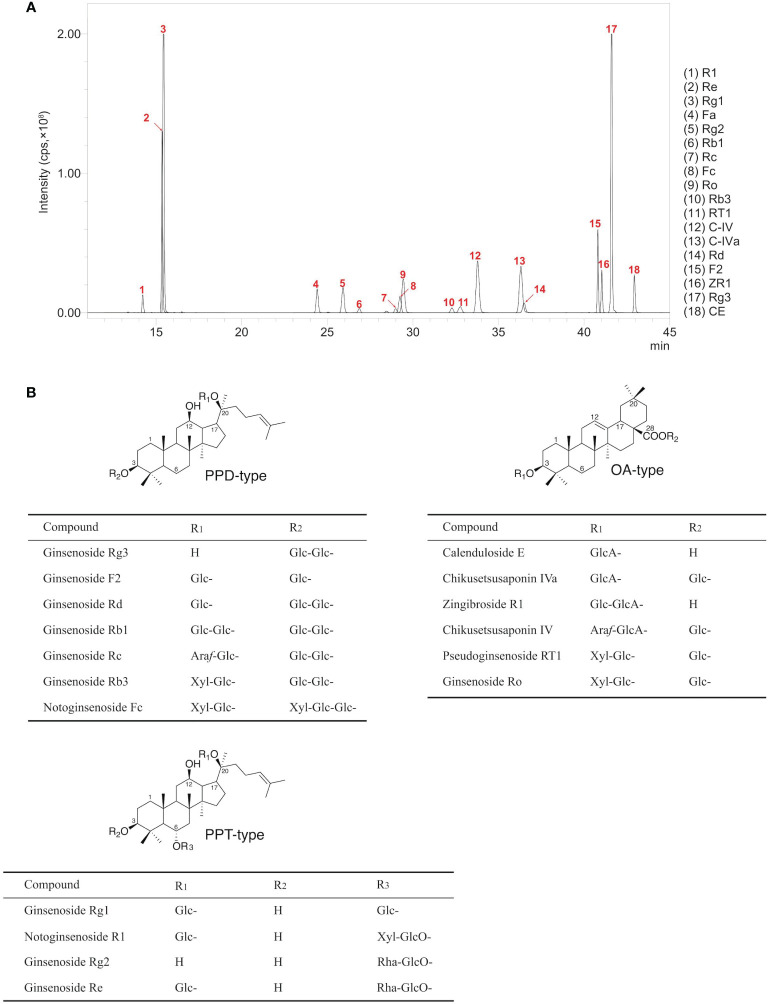
LC-MS result of ginsenoside extracts from four tissues of *P. japonicus*. **(A)** Total ion chromatogram of 18 ginsenosides. Notoginsenoside R1 (R1, PPT-type); Ginsenoside Re (Re, PPT-type); Ginsenoside Rg1 (Rg1, PPT-type); Notoginsenoside Fa (Fa, PPD-type); Ginsenoside Rg2 (Rg2, PPT-type); Ginsenoside Rb1 (Rb1, PPD-type); Ginsenoside Rc (Rc, PPD-type); Notoginsenoside Fc (Fc, PPD-type); Ginsenoside Ro (Ro, OA-type); Ginsenoside Rb3 (Rb3, PPD-type); Pseudoginsenoside RT1 (RT1, OA-type); Chikusetsusaponin IV (C-IV, OA-type); Chikusetsusaponin IVa (C-IVa, OA-type); Ginsenoside Rd (Rd, PPD-type); Ginsenoside F2 (F2, PPD-type); Zingibroside R1 (ZR1, OA-type); Ginsenoside Rg3 (Rg3, PPD-type); Calenduloside E (CE, OA-type). **(B)** Chemical structures of 18 ginsenosides.

The UPLC-MRM-MS method was applied to quantify the contents of ginsenosides in rhizomes, stems, leaves, and lateral roots of *P. japonicus* growth in August from 1 to 3 years. In this study, 18 kinds of ginsenosides mainly distributed in *P. japonicus* subdivided into three aglycone types (OA, PPD, and PPT type) were chosen to determine for more comprehensively exploring relevant key genes in the biosynthetic pathway of ginsenosides (**Supplementary Table S3**). As shown in the total ion chromatography (TIC) of rhizomes (**Figure 1**), eighteen peaks were identified by comparing the retention times of corresponding references. All coefficients of determination (*R*) are higher than 0.998 intending good linearities over the standard curve lines (**Supplementary Table S2**). Therefore, the contents of ginsenosides in different samples were further calculated using the corresponding standard curve.

**Figure 2:**
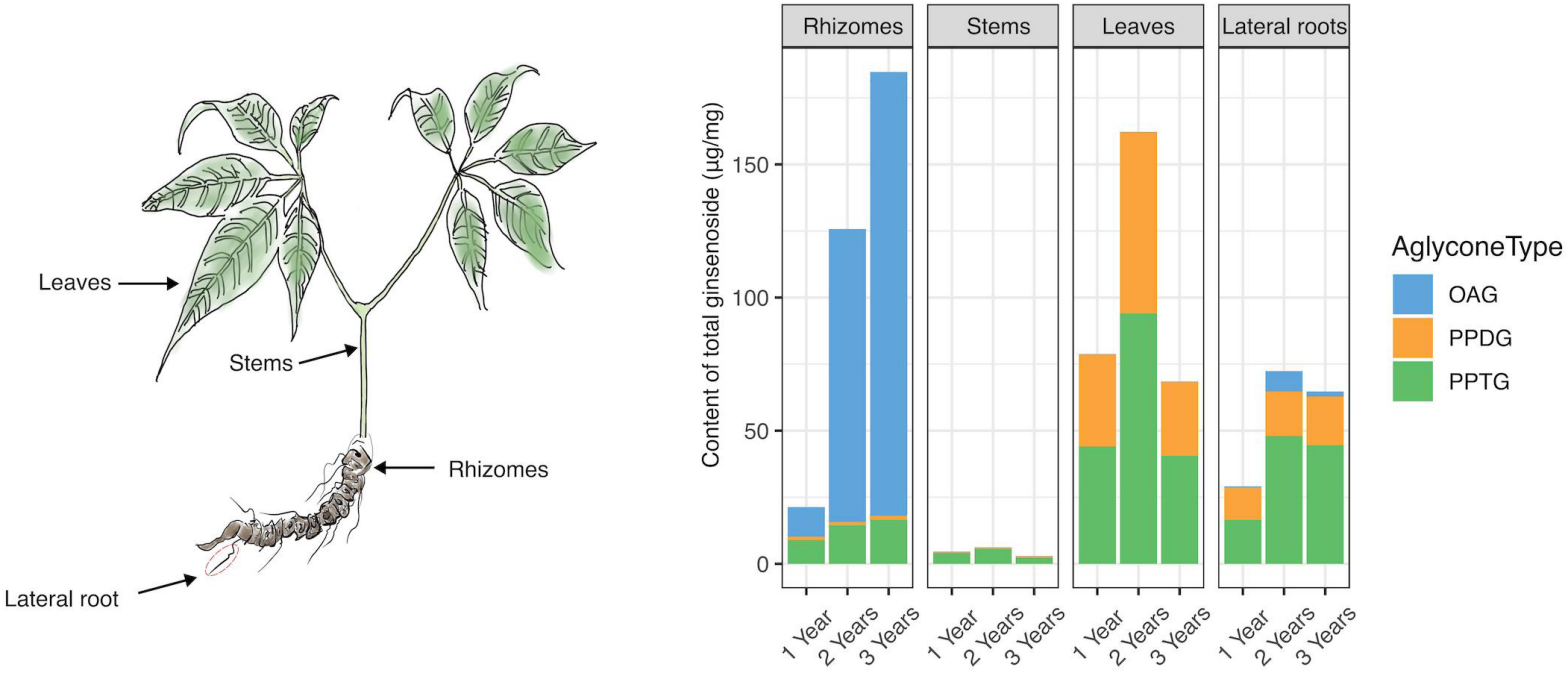
The cumulative histogram plotting for the total content of ginsenosides measured in different *P. japonicus* samples. Eighteen investigated ginsenosides in four plant tissues from *P. japonicus* over the early growth stage. Data represent the mean of three biological replicates. “OAG” represents OA-type ginsenosides, containing 6 ginsenosides. “PPDG” is PPD-type ginsenosides, containing 8 ginsenosides. “PPTG” represents PPT-type ginsenosides, containing 4 ginsenosides.

As shown in **Figure 2**, it was suggested that there were significantly disparate metabolite profiles in four different tissues of *P. japonicus*. In rhizomes, the total content of ginsenosides (TCG) was positively correlated with the growing age. The increase of TCG was mainly caused by the growth of OA-type ginsenoside (OAG) in the proportion and content of TCG. In stems, ginsenosides had relatively poor diversity and were mainly composed of PPT-type ginsenosides (PPTG). TCG in stems was the lowest among four different tissues of *P. japonicus*. In leaves, ginsenosides were mainly composed of PPTG and PPD-type ginsenosides (PPDG), and the content of PPTG was slightly higher than PPDG. In lateral roots, the content of PPTG was the highest, followed by PPDG, and OAG was the least.

**Figure 3:**
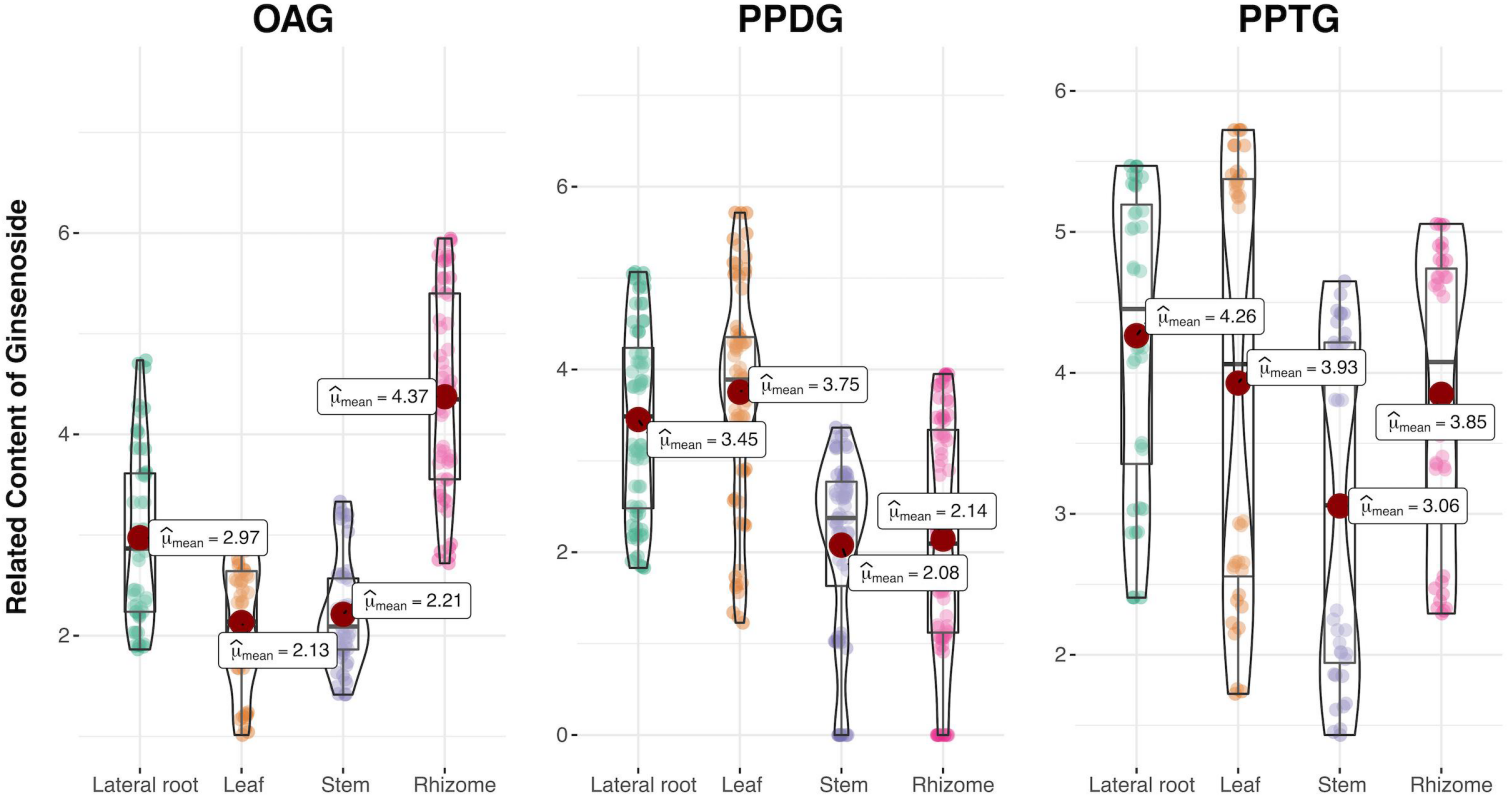
Violin plots representing the related content of non-additively accumulated ginsenosides in three types from four tissues of *P. japonicus*. R ‘ggstatsplot’ package was applied and all figures include a boxplot as well as a violin plot showing the data distribution. Each box extends from the lower to the upper quartile, and the line in each box represents the mean. The outer line extends to the minimum and maximum data points. The mean represents the accumulation of a certain type of ginsenoside in different tissues.

Moreover, it was shown that ginsenosides in three types have various distribution preferences in **Figure 2**. Then a violin plot with the content of every compound as a data point was further performed to accurately analyze the distribution preferences by reducing the influence of the single compound content on the overall category. The compounds in different tissues were grouped according to the type of ginsenoside (OA, PPD, and PPT-type), and the content data was multiplied by 10000 and added 1 to make a log_10_ times change. Then statistical comparisons between tissue groups were carried out using Dunn’s test implementation in the R ‘ggstatsplot’ package (Patil, 2021). The mean value was used as the criterion to analyze the distribution of three kinds of ginsenosides in four different tissues of *P. japonicus*. As shown in **Figure 3**, OAG was mainly distributed in rhizomes and least in leaves, while PPDG was most distributed in leaves and least in stems. PPTG was mainly distributed in lateral roots and least in stems. The specific metabolic distribution preferences in different tissues of *P. japonicus* are dependent on the expression regulation of a set of key genes in the biosynthetic pathways of ginsenosides. Therefore, the contents of three types of ginsenosides in each sample were further compared with verify genes of interest (GOI) through differential gene expression analysis. Finally, three pairs of samples with the most significant differences were obtained in the production of three types of ginsenosides (**Supplementary Figure S1**).

### Transcriptomic analysis and annotation

Transcriptome-guided genetic analysis is a useful method to reveal molecular mechanisms in plant phenotypes, and it is also very effective for elucidating plant biosynthetic pathways (Nett et al., 2021). In this study, 12 samples collected from the rhizomes, stems, leaves and lateral roots of *P. japonicus* in August from 1 to 3 years were set for Illumina sequencing. All the clean reads were pooled together and *de novo* assembled into transcripts by Trinity software. Subsequently, unigenes were constructed based on these transcripts. All the quantitative parameters of this assembled transcriptome were summarized in **Supplementary Table S4**. It is feasible to perform gene differential expression analysis on plant samples lacking a reference sequenced genome by assembling short-read fragments from Illumina sequencing, this method may result in the incorrect transcriptomic analysis due to fragmented and misassembled contigs, leading to deficiency of biological information and inaccuracy analysis results. To avoid the above technical limitations of *de novo* transcriptome assembly, PacBio Single-Molecule Real-time (SMRT) sequencing was utilized to obtain full-length complementary DNA (cDNA) reads for RNA isolated from *P. japonicus* tissues. Then Illumina sequencing with libraries from multiple plant tissues were utilized to quantify relative expression within this transcriptome.

To obtain a full-scale functional annotation of *P. japonicus* transcriptome, all full-length transcripts were searched using BLASTX against five databases: RefSeq non-redundant proteins (NR), Kyoto Encyclopedia of Genes and Genomes (KEGG), Gene Ontology (GO), Swiss-Prot, Pfam. In total, 279,109 transcripts (37.2%) had the most significant BLAST matches with known sequences in all databases. Detailed information was listed in **Supplementary Table S5**, including the percentage and number of successfully annotated transcripts in each database. To reveal the specific physiological function of each transcript, enrichment analysis was performed for all transcripts successfully annotated in the GO database and KEGG database (**Supplementary Figure S2**). A total number of 45,043 transcripts were annotated in the GO database and were classified into three main categories as follows: biological process (BP), molecular function (MF) and cellular component (CC). In CC category, “cell” and “cell part” were the largest subcategory with similar numbers, while “metabolic process” was the predominant subcategory in BP category. In MF category, “binding” was the largest subcategory and followed by “catalytic reaction”. These results indicated that there were vigorous metabolic activities and enzyme-catalyzed reactions in the early growth stage of *P. japonicus*. In KEGG categories, the most enriched subcategory was “Metabolism” (12,163), and up to 144 transcripts were annotated as being related to the metabolism of terpenoids and polyketides, showing that there were active physiological activities related to the biosynthesis of ginsenosides in the early growth stage of *P. japonicus*.

### Dataset integration and DEGs identification

**Figure 4:**
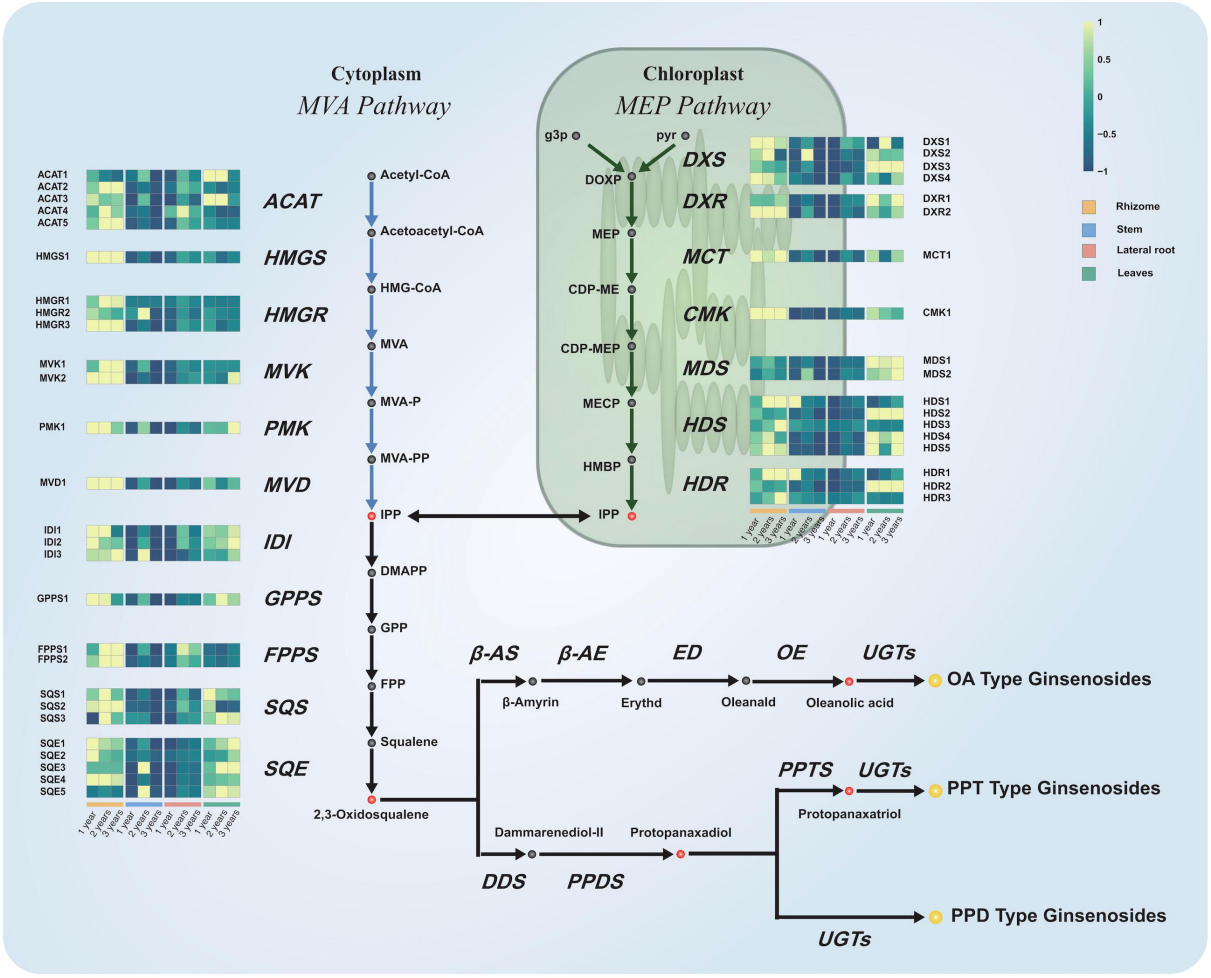
Expression profiles of genes in the ginsenoside biosynthetic pathway. Schematic representation of ginsenosides biosynthetic pathway and the expression profile of those transcripts of the *P. japonicus* species which coding for the enzymes involved. The grey, red, and yellow circles marked the proposed intermediates, precursors, and ginsenosides in the biosynthetic pathway of ginsenosides. The enzymes (acronyms shown in italics) involved in the ginsenosides biosynthetic pathway and their corresponding orthologs identified in the *P. japonicus* transcriptome were represented. Expression profiles represent the abundance of the transcripts (FPKM) in each tissue from different growth years.

Principal component analysis (PCA) was performed on the RNA-seq data to detect outlier samples. As shown in **Supplementary Figure S3**, there was no outlier detected in PCA. The gene expression pattern of different tissues was specific and consistent with the conclusion obtained from the metabolite analysis. As mentioned before, ginsenosides were originally derived from MVA and MEP pathways and finally modified by UGTs and CYPs. Hence, 45 genes were screened involved in MVA pathway, MEP pathway and triterpene synthetic pathway based on the genetic annotation of transcripts (Hou et al., 2021). Furthermore, a heatmap of gene expression grouped by plant tissues was plotted to figure out the differential expression between samples. In **Figure 4**, the grid corresponding to higher gene expression was displayed in brighter green, and the corresponding data are presented in **Supplementary Table S6**. The expression abundance of 45 candidate genes had very significant tissue specificity. The order of their expression levels in different tissues of *P. japonicus* were rhizomes, leaves, lateral roots and stems from high to low. This result was also highly consistent with the results of previous metabolite analysis. Therefore, it was important to use the conjoint analysis strategy of transcriptome and metabolite data to identify potential downstream genes for the biosynthesis of ginsenosides.

**Figure 5:**
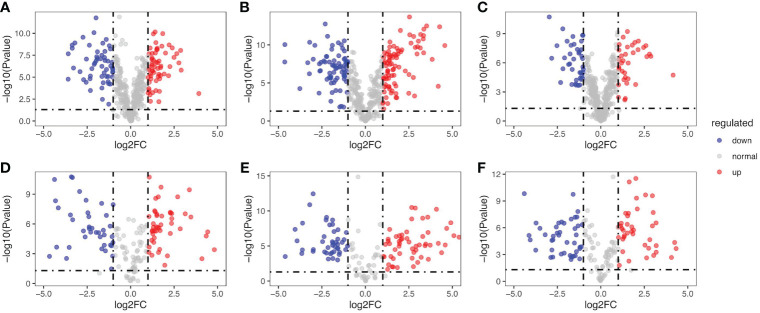
Transcriptome-based quantitative profiling of CYPs and UGTs in the differential samples. Volcano plot of *p*-value (log2 scale) vs fold-change (FC, positive if DEGs are higher expressed in samples with higher ginsenosides accumulation). The red and green points denote upregulated DEGs and downregulated DEGs in samples with higher ginsenosides accumulation, respectively. The grey points denote genes with no significant difference in expression between samples with higher ginsenosides accumulation and samples with lower ginsenosides accumulation. Differential expression analysis of UGTs: **(A)** 3yr_R vs 3yr_L, **(B)** 2yr_L vs 2yr_S, **(C)** 2yr_LR vs 3yr_S. Differential expression analysis of CYPs: **(D)** 3yr_R vs 3yr_L, **(E)** 2yr_LR vs 3yr_S, **(F)** 2yr_LR vs 3yr_S.

Among the cascade reactions of ginsenoside synthesis, the skeleton decorations catalyzed by CYPs and UGTs had been already identified as the rate-limiting steps (Hou et al., 2021). The contents of ginsenosides in *P. japonicus* were related to the expression levels of CYPs and UGTs. To analyze the expression of CYPs and UGTs in obtained three pairs of samples, 159 genes annotated as CYPs and 419 genes as UGTs were quantified and their expression levels were further compared. These genes were then ranked in corresponding volcano plots as log_2-_ fold change (High-content individual/Low-content individual) against statistical significance (-log^10^ *p*-value). Moreover, genes with both (log^2-^) ratios higher than 1.00 and a *p*-value less than 0.05 were considered significant hits. As shown in **Figure 5**, there were 60, 87, and 35 UGTs were identified as significantly different hits in groups A, B, and C. In addition, there were 47, 55, 41 CYP450 were determined to be significantly different hits in groups D, E, and F, respectively. These results indicated that there were indeed differential expressions of CYPs and UGTs in selected samples, which might be responsible for the specific metabolite profiles of different *P. japonicus* tissues. This also provided an important basis for subsequent correlation analysis between gene abundance and ginsenoside content.

### Correlation analysis of transcripts related to ginsenosides

**Figure 6:**
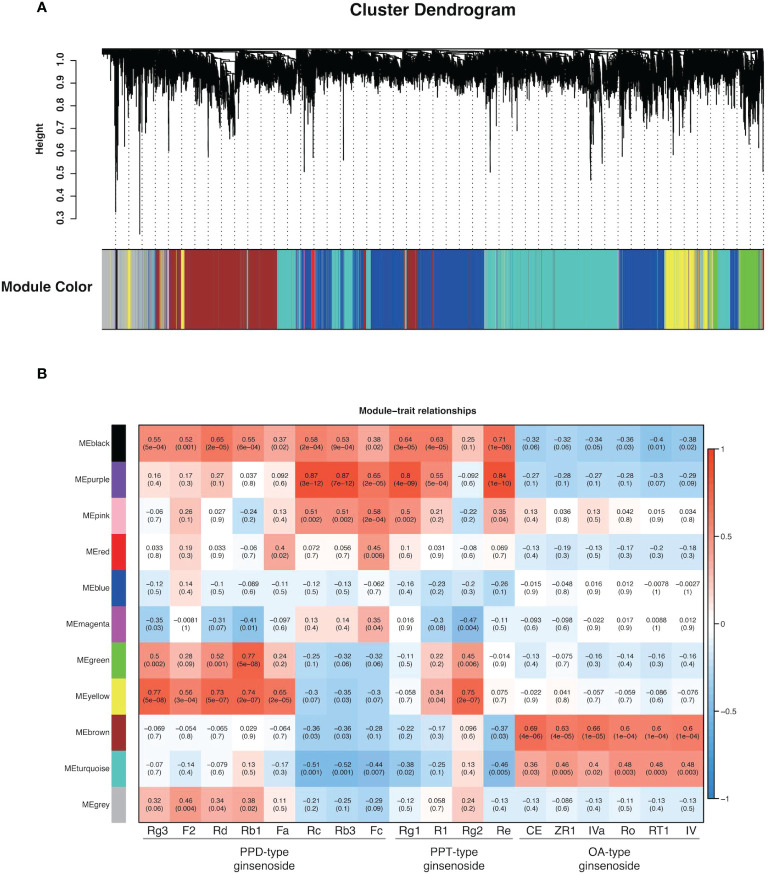
WGCNA of 17,885 genes with ginsenoside content data. **(A)** The hierarchical cluster tree indicates the co-expression modules identified by WGCNA. Each leaf in the tree represents one gene, and major branches labeled with different colors constitute 11 modules. **(B)** Module−content correlations. The correlation coefficient between the module and sample is described by the color of each cell at the row−column intersection, and the color scale on the right shows module-trait correlation from -1 (blue) to 1 (red). Numbers within cells represent correlations and *p*-values (in parenthesis).

**Figure 7:**
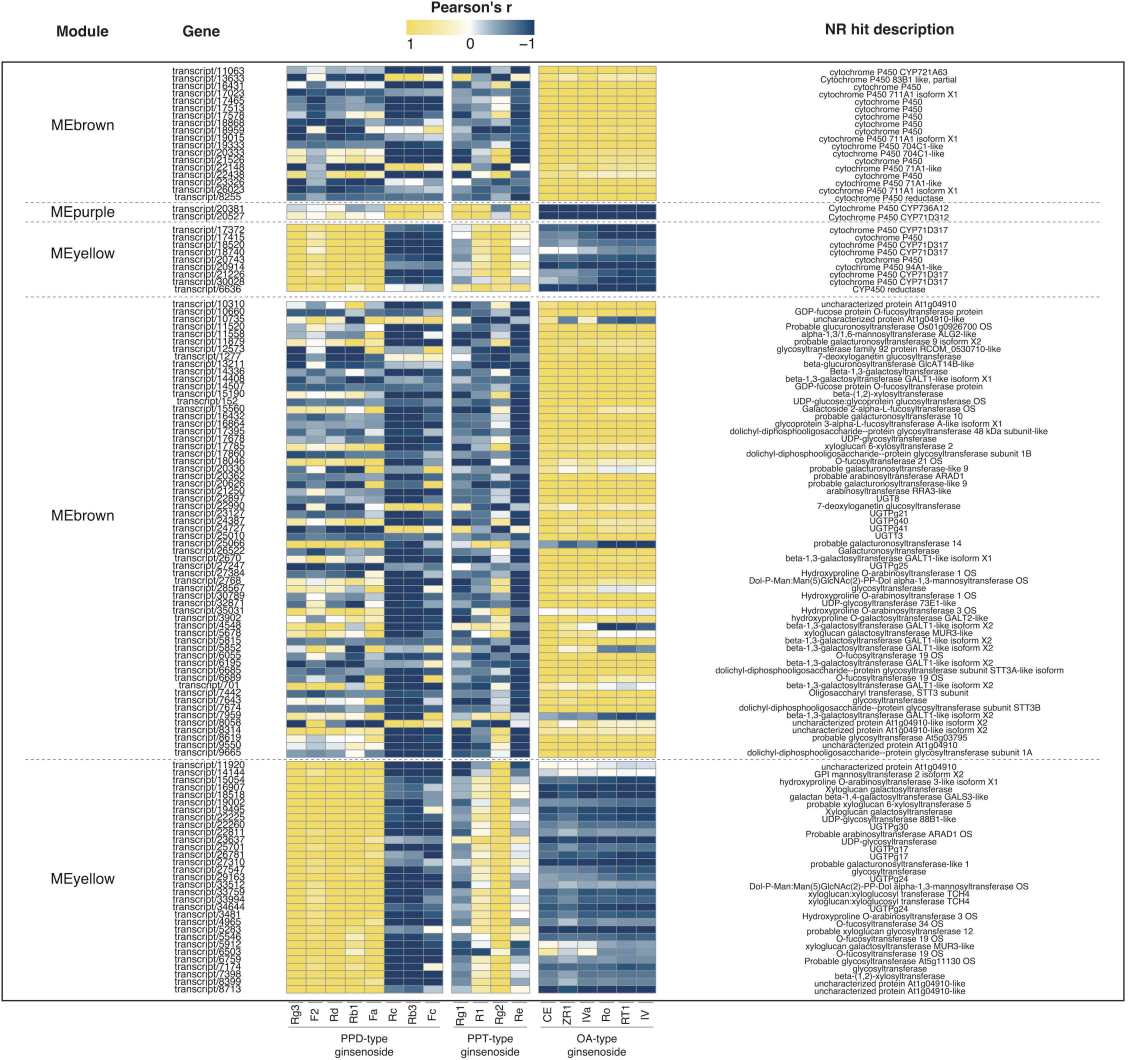
Pearson correlation analysis of target transcripts expression patterns and ginsenoside contents in different samples. Heatmap to present the Pearson’s correlation of CYPs and UGTs annotated transcripts from 5 selected modules with investigated ginsenosides.

Weighted Correlation Network Analysis (WGCNA) is a systematic biological approach for describing the patterns of genes associated with different samples, and it is frequently to identify gene modules and hub genes that are highly related to specific phenotypes. In the process of WGCNA, totally 17,885 genes (FPKM > 10) were used in all biological samples. An unsigned network was constructed using the transcriptome dataset. All transcripts with the same expression pattern were co-localized into 10 modules (MEblack, MEpurple, MEpink, MEred, MEblue, MEmagenta, MEgreen, MEyellow, MEbrown, and MEturquoise) and transcripts without significantly identical expression patterns into MEgrey (**Figure 6A**). MEturquoise had the most transcripts with 5,321, and Mepurple had the fewest transcripts with 33. It was worth noting that WGCNA provided important clues for identifying gene modules associated with the synthesis of 18 ginsenosides in three types. As shown in **Figure 6B**, oleanane-type ginsenosides and dammarane-type ginsenosides had completely different specific genes. OA-type ginsenosides had a strong positive correlation with MEbrown, while PPD-type and PPT-type ginsenosides had a strong positive correlation with MEpurple and MEyellow, respectively. The correlation results between contents of ginsenosides and expression abundance of CYPs and UGTs from MEbrown, Mepurple, and MEyellow *via* pearson correlation analysis revealed that the candidate CYPs and UGTs in each module were consistent with the expression pattern of the responding module and were also closely related to the contents of target ginsenosides (**Figure 7**), and the data are shown in **Table S7**.

### Correlation analysis of protein related to ginsenosides

**Figure 8:**
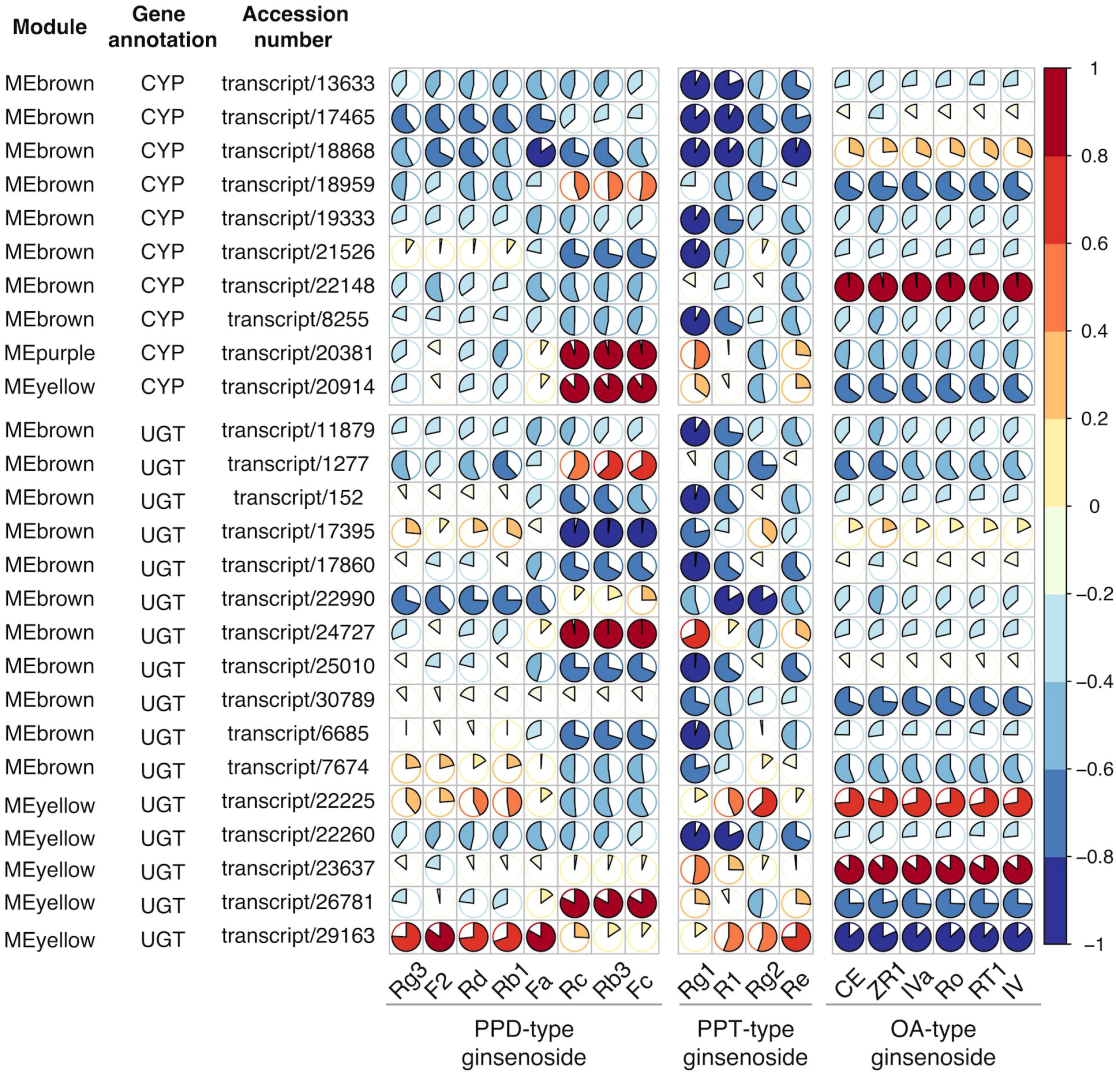
Pearson correlation analysis of target protein expression patterns and ginsenoside contents in different tissues. Based on integrated transcriptome and proteome analysis, 26 transcripts/proteins were identified. Pie plot to present the Pearson’s correlation of selected CYPs and UGTs annotated transcripts/proteome with investigated ginsenosides. The area of circles corresponds to correlation coefficient (R) values, and colors indicate whether a correlation is negative or positive.

Despite of considerable sensitivity, the transcriptome does not clearly represent the true phenotype because mRNA analysis fails to capture regulatory processes or post-transcriptional modifications that may affect the amounts of active proteins. Proteomics lacks the sensitivity to detect low-abundance proteins and are limited to identify novel proteomics arising from alternative splicing. However, integrated analysis of the transcriptome and proteome may provide complementary information to draw more informative conclusions (Kumar et al., 2016; Liu et al., 2019). Encouraged by the compound-correlated expression of transcripts in the transcriptome analysis, proteomic analysis was performed for three-year-old *P. japonicus* samples. After comparatively analyzing the transcriptome and proteome data, the same 10 CYPs and 16 UGTs were screened in both the transcriptome and proteome sequencing. Then the correlation was further analyzed between their protein expression and the content of 18 ginsenosides in each sample, as shown in **Table S8**. The expression levels of candidate proteins were all strongly correlated with ginsenosides content (correlation coefficient>0.8), except for one CYPs transcript (transcript/18959) and four UGTs transcripts (transcript/1277, transcript/30789, transcript/7674, and transcript/22225) (**Figure 8**). This result suggested that the target transcripts might be involved in the biosynthesis of ginsenosides and associated regulation at the protein expression level. The catalytic functions of target enzymes were preliminarily predicted through protein-compound correlation analysis. It was speculated that proteins negatively correlated with a certain ginsenoside may be involved in its downstream metabolites production, while the protein positively correlated with a certain ginsenoside may be involved in the synthesis of the ginsenoside or upstream metabolites.

### Phylogenetic analysis

**Figure 9:**
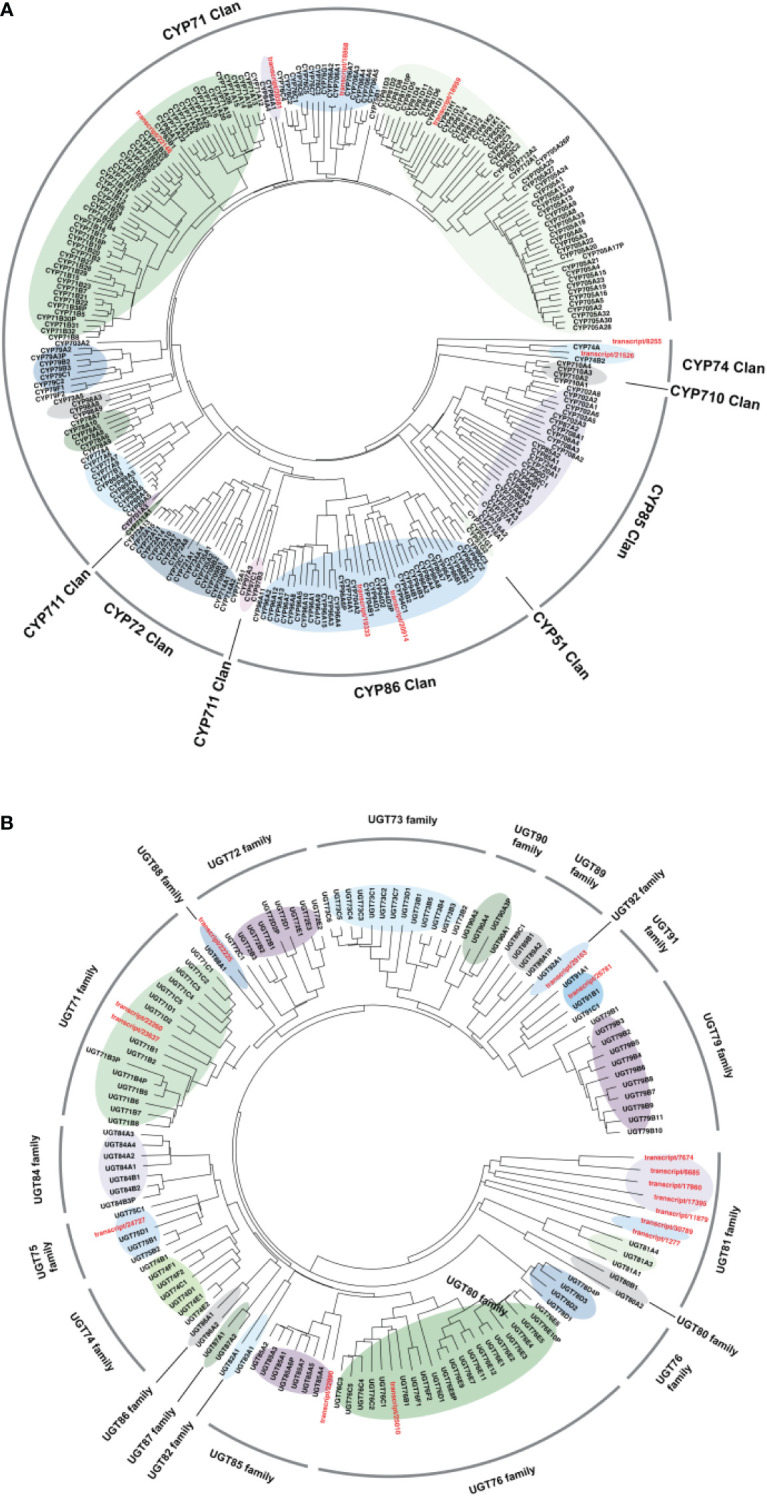
Phylogenetic tree of candidate genes with the representative. **(A)** CYP450 and **(B)** UGT family members. MEGAX software was used to align amino acid sequences of target genes and draw the Neighbor-Joining tree.

To further characterize the functions of candidate CYPs transcripts and UGTs transcripts identified in the above work, phylogenetic analysis was carried out for 254 CYPs and 122 UGTs annotated genes in the *Arabidopsis* genome obtained from http://www.p450.kvl.dk/index.shtml (Liu et al., 2019) together with all corresponding candidate genes, and grouped genes in different clades as described (**Figure 9**).

Cytochrome P450s constitute a superfamily of highly differentiated sequences, the largest family of enzymes in plant metabolism (Paquette et al., 2000). The classification of CYPs is based on their amino acid homology. If the homology of more than 40%, CYPs will be classified as the same gene family. If the homology of more than 55%, CYPs will be categorized as the same subfamily. Currently, 127 CYPs families have been discovered in plants and were grouped into 11 clans according to their branches on the phylogenetic tree (Nelson and Werck-Reichhart, 2011). It was reported that CYPs related to triterpenoid structural modification were mainly distributed in the CYP51H subfamily of the CYP51 clan, CYP71A, CYP71D, CYP81Q, CYP93E, and CYP705A subfamily of CYP71 clan, CYP72A, CYP714E and CYP749A subfamily of CYP72 clan, CYP94N subfamily of CYP86 clan, CYP85 CYP87D, CYP88D, CYP88L, CYP708A, CYP716A, CYP716C, CYP716E, CYP716S, CYP716U, CYP716Y, CYP90B, and CYP90G subfamily (Bak et al., 2011). According to the phylogenetic analysis (**Figure 9A**), four unigenes (transcript/22148, transcript/20381, transcript/18868, and transcript/18959) were clustered in the CYP71 clan that was found to participate in triterpene modifications. And two unigenes (transcript/8255, transcript/21526) were clustered in the CYP74 clan related to the regulation of jasmonate acid pathway. In addition, two unigenes (transcript/20914 and transcript/19333) were clustered in the CYP86 clan involved in fatty acid oxygenation (Zhu et al., 2019). As shown in **Figure 9B**, most of candidate UGTs transcripts were classified into the UGT81 family based on phylogenetic analysis, while others were classified into the UGT76, UGT85, UGT75, UGT71, UGT88, UGT92, UGT91 families, respectively. It was worth noted that studies had already identified glycosyltransferases clustered in UGT85 (Scholz et al., 2010), UGT71 (Zhai et al., 2016), and UGT91 (Shibuya et al., 2010) families were responsible for the glycosylation of triterpenoid saponins or aglycones. According to the phylogenetic analysis, 4 CYPs (transcript/22148, transcript/20381, transcript/18868, and transcript/18959) and 4 UGTs (transcript/22990, transcript/22260, transcript/23637, and transcript/26781) may be involved in triterpenoid saponins biosynthesis. Further investigations will be required to determine the role of these potential transcripts in ginsenosides biosynthesis.

Ginsenosides in *P. japonicus* possess high nutritional and medicinal values, but there is a poor understanding of its biosynthesis and accumulation. In this study, the dynamics of ginsenosides accumulation were systematically investigated. The transcriptome and proteome from four tissues of *P. japonicus* in early growth stages were sequenced and annotated. Twenty enzymes were identified in the ginsenosides biosynthetic pathway. Further research such as heterologous expression and studies of substrate specificity or regulation of enzyme activity will shed light on our understanding of the ginsenosides synthetic pathway and lay a foundation for molecular breeding.

## Conclusion

This study first revealed and analyzed the temporospatial distribution and genes involved in the biosynthesis of ginsenosides in *P. japonicus*. There were significantly different metabolic profiles in the four different tissues of *P. japonicus*. OA-type ginsenosides are mainly distributed in rhizomes, PPD-type ginsenosides are mainly distributed in leaves, and PPT-type ginsenosides are mainly distributed in lateral roots. To reveal the saponins synthesis and distribution pattern in *P. japonicus* a combined transcriptomic and proteomic analysis was applied. In this study, integration of the transcriptome and proteome profiles uncovered several candidate transcripts/proteins that might be involved in ginsenoside biosynthesis. A total of 750,245 transcripts and 3,482 protein groups were identified in *P. japonicus*. Based on transcriptome data, 29 CYPs, 92 UGTs genes and three gene modules related to ginsenoside biosynthesis through differential expression analysis and correlation analysis. Then the corresponding 8 CYPs and 15 UGTs proteins were found in the proteome and classified and predicted their functions. This study provides extensive new information on the transcriptome and proteome of *P. japonicus*. Analysis of this resource has enabled us to understand the synthesis mechanism of ginsenosides at the molecular level, which provides essential information on the metabolic regulation for *P. japonicus* cultivation and breeding.

## Data availability statement

The original contributions presented in the study are publicly available. This data can be found here: NCBI, PRJNA885251 and ProteomeXchange, PXD038579.

## Author contributions

ZW and RW conceived and designed the experiments. CH, PL, XY, and TN performed the experiments. CH, PL, SZ, and LY analyzed the data. CH and RW wrote the manuscript. All authors contributed to the article and approved the submitted version.
