# Supplementary material for: Integrated transcriptome and proteome analyses reveal candidate genes for ginsenoside biosynthesis in Panax japonicus C. A. Meyer
Source: Front Plant Sci. 2023 Jan 9;13:1106145. doi: 10.3389/fpls.2022.1106145 (PMC9868605; doi:10.3389/fpls.2022.1106145)
Supplement: Supplementary file 1 [file DataSheet_1.docx]

Supplementary Material

**Integrated transcriptome and proteome analyses reveal candidate genes for ginsenoside biosynthesis in *Panax japonicus* C. A. Meyer**

**Chaokang Huang^1^, Pengfei Li^1^, Xiaolin Yang^1^, Tengfei Niu^1^, Shujuan Zhao^1,2^ , Li Yang^1,2^, Rufeng Wang^1,2*^, and Zhengtao Wang^1,2*^**

^1^ The SATCM Key Laboratory for New Resources and Quality Evaluation of Chinese Medicines, Institute of Chinese Materia Medica, Shanghai University of Traditional Chinese Medicine, Shanghai 201203, P.R. China

^2^ The MOE Key Laboratory for Standardization of Chinese Medicines, Institute of Chinese Materia Medica, Shanghai University of Traditional Chinese Medicine, Shanghai 201203, P.R. China.

*Corresponding authors. Tel.: +86-21-5132-2495; Fax: +86-21-5132-0840; E-mails: wrffrw0801@ shutcm.edu.cn (R.F. Wang).

**Supplementary Table S1.** The optimized MRM parameters and transitions for each analyst in UPLC-MRM-MS.

| Compound | Precusor m/z | Product m/z | Dwell Time (msec ) | Q1 Pre Bias (V) | CE (V) | Q3 Pre Bias (V) |
| --- | --- | --- | --- | --- | --- | --- |
| Notoginsenoside R1 | 931.60 | 637.45 | 100 | 26.0 | 40.0 | 32.0 |
| Ginsenoside Re | 991.50 | 945.40 | 100 | 28.0 | 25.0 | 26.0 |
| Ginsenoside Rg1 | 845.60 | 799.50 | 100 | 20.0 | 26.0 | 22.0 |
| Notoginsenoside Fa | 1239.60 | 1107.45 | 100 | 46.0 | 50.0 | 32.0 |
| Ginsenoside Rb1 | 1107.75 | 945.55 | 100 | 32.0 | 45.0 | 26.0 |
| Ginsenoside Rg2 | 783.60 | 475.35 | 100 | 22.0 | 38.0 | 23.0 |
| Ginsenoside Ro | 955.50 | 793.35 | 100 | 26.0 | 45.0 | 22.0 |
| Ginsenoside Rc | 1077.75 | 945.50 | 100 | 30.0 | 43.0 | 26.0 |
| Notoginsenoside Fc | 1209.85 | 1077.60 | 100 | 34.0 | 48.0 | 30.0 |
| Ginsenoside Rb3 | 1077.70 | 783.35 | 100 | 30.0 | 46.0 | 22.0 |
| Pseudoginsenoside RT1 | 925.40 | 569.25 | 100 | 34.0 | 46.0 | 28.0 |
| Chikusetsusaponin IV | 925.40 | 569.30 | 100 | 22.0 | 41.0 | 28.0 |
| Chikusetsusaponin IVa | 793.50 | 631.30 | 100 | 22.0 | 42.0 | 30.0 |
| Ginsenoside Rd | 945.55 | 783.45 | 100 | 36.0 | 38.0 | 22.0 |
| Ginsenosdie F2 | 829.55 | 783.50 | 100 | 30.0 | 23.0 | 22.0 |
| Zingibroside R1 | 793.40 | 613.30 | 100 | 22.0 | 45.0 | 30.0 |
| Ginsenoside Rg3 | 783.55 | 621.40 | 100 | 30.0 | 32.0 | 30.0 |
| Calenduloside E | 631.40 | 455.20 | 100 | 30.0 | 41.0 | 22.0 |

**Supplementary Table S2.** Calibration curves and detection limit of 18 ginsenosides in *P. japonicus*.

| Compound | Regression Equations | *R*^2^ | Linear Ranges (μg/mL) |
| --- | --- | --- | --- |
| Notoginsenoside R1 | *y*=204507*x* - 6953.5 | 0.9992 | 0.01~5 |
| Ginsenoside Re | *y*=2×10^6^*x* - 432381 | 0.9997 | 0.002~40 |
| Ginsenoside Rg1 | *y*=1×10^6^*x* - 116369 | 0.9997 | 0.5~20 |
| Notoginsenoside Fa | *y*=282486*x* - 7586.1 | 0.9983 | 0.006~2 |
| Ginsenoside Rb1 | *y*=41102*x* - 718.2 | 0.9999 | 0.02～10 |
| Ginsenoside Rg2 | *y*=545880*x* - 7477.6 | 0.9999 | 0.008~2 |
| Ginsenoside Ro | *y*=625166*x* - 62261 | 0.9999 | 0.01~40 |
| Ginsenoside Rc | *y*=62674*x* - 16115 | 0.9992 | 0.5~20 |
| Notoginsenoside Fc | *y*=181493*x* - 813.03 | 1 | 0.005~20 |
| Ginsenoside Rb3 | *y*=57566*x* - 3599.9 | 0.9987 | 0.2~10 |
| Pseudoginsenoside RT1 | *y*=2×10^6^*x* - 88039 | 0.9999 | 0.002~20 |
| Chikusetsusaponin IV | *y*=2×10^6^*x* - 247691 | 0.9996 | 0.02~20 |
| Chikusetsusaponin IVa | *y*=1×10^6^*x* - 29301 | 0.9999 | 0.002~10 |
| Ginsenoside Rd | *y*=158536*x* - 13020 | 1 | 0.1~20 |
| Ginsenosdie F2 | *y*=2×10^6^*x* - 32608 | 0.9999 | 0.02~10 |
| Zingibroside R1 | *y*=669980*x* + 4443 | 1 | 0.008~5 |
| Ginsenoside Rg3 | y=380861*x* - 1087.2 | 0.9983 | 0.002~0.2 |
| Calenduloside E | y=787866*x* - 3850.6 | 0.9988 | 0.001~0.2 |

**Supplementary Table S3.** Content of diverse ginsenosides in *P. japonicus* samples.

| Aglycone Type | Compound | Rhizome (μg/mL) | | | Leaf (μg/mL) | | | Stem (μg/mL) | | | Lateral root (μg/mL) | | |
| --- | --- | --- | --- | --- | --- | --- | --- | --- | --- | --- | --- | --- | --- |
|  |  | 1 year | 2 years | 3 years | 1 year | 2 years | 3 years | 1 year | 2 years | 3 years | 1 year | 2 years | 3 years |
| OA Type | Ginsenoside Ro | 3.66062 | 59.62224 | 84.08250 | 0.03494 | 0.04145 | 0.02669 | 0.01958 | 0.03841 | 0.04061 | 0.21126 | 5.04377 | 1.07129 |
|  | Pseudoginsenoside RT1 | 0.05670 | 0.24628 | 0.35699 | 0.00680 | 0.00606 | 0.00620 | 0.01514 | 0.00931 | 0.01762 | 0.00767 | 0.02361 | 0.01066 |
|  | Chikusetsusaponin IV | 6.04656 | 35.75504 | 53.16972 | 0.05399 | 0.04020 | 0.02127 | 0.18704 | 0.13535 | 0.15698 | 0.11364 | 1.81699 | 0.41256 |
|  | Chikusetsusaponin IVa | 0.60929 | 12.59625 | 25.40694 | 0.00713 | 0.00837 | 0.00467 | 0.00846 | 0.01057 | 0.01474 | 0.00790 | 0.06262 | 0.01951 |
|  | Zingibroside R1 | 0.67719 | 1.67684 | 3.07610 | 0.08233 | 0.08711 | 0.08141 | 0.00498 | 0.00964 | 0.00716 | 0.07836 | 0.72295 | 0.39252 |
|  | Caleouloside E | 0.07457 | 0.19476 | 0.54316 | 0.00158 | 0.00143 | 0.00100 | 0.00329 | 0.00536 | 0.00252 | 0.01585 | 0.02822 | 0.01717 |
| PPD Type | Notoginsenoside Fa | 0.00388 | 0.00366 | 0.01220 | 0.02015 | 0.03675 | 0.07903 | 0.01619 | 0.00723 | 0.01258 | 0.05234 | 0.02641 | 0.14951 |
|  | Ginsenoisde Rb1 | 0.80284 | 0.66496 | 0.87542 | 0.30725 | 0.80020 | 0.28914 | 0.02351 | 0.05911 | 0.04255 | 7.97175 | 11.37492 | 9.93182 |
|  | Ginsenoside Rc | - | - | - | 0.95527 | 1.78918 | 0.54631 | - | - | - | 0.12856 | 0.12746 | 0.10609 |
|  | Notoginsenoside Fc | 0.00146 | 0.00140 | 0.00356 | 2.17861 | 11.12060 | 11.00242 | 0.01664 | 0.01647 | 0.02369 | 0.01437 | 0.01856 | 0.03135 |
|  | Ginsenoside Rb3 | 0.11875 | 0.18431 | 0.19067 | 26.80615 | 51.75887 | 14.77373 | 0.05724 | 0.06775 | 0.07483 | 0.64854 | 1.06088 | 1.20346 |
|  | Ginsenoside Rd | 0.29732 | 0.29773 | 0.46675 | 2.39736 | 1.98880 | 0.71275 | 0.05087 | 0.03937 | 0.03934 | 2.60541 | 3.35863 | 5.25964 |
|  | Ginsenoside F2 | 0.07888 | 0.01325 | 0.01752 | 1.85386 | 0.53268 | 0.40968 | 0.18225 | 0.18568 | 0.14104 | 0.74937 | 0.66873 | 1.50196 |
|  | Ginsenoside Rg3 | 0.00116 | 0.00078 | 0.00107 | 0.00526 | 0.00402 | 0.00184 | 0.00107 | 0.00093 | 0.00096 | 0.00682 | 0.00840 | 0.01539 |
| PPT Type | Notoginsenoside R1 | 0.02404 | 0.02043 | 0.03278 | 0.04124 | 0.08557 | 0.04535 | 0.01024 | 0.01774 | 0.00707 | 0.02541 | 0.10788 | 0.07376 |
|  | Ginsenoside Re | 4.72400 | 6.27903 | 4.77542 | 21.51199 | 52.82091 | 21.37369 | 2.63578 | 3.61555 | 1.63940 | 10.65579 | 25.09569 | 29.18477 |
|  | Ginsenoside Rg1 | 3.87179 | 7.96823 | 11.29946 | 22.46187 | 41.05772 | 19.09917 | 1.35856 | 1.90092 | 0.64151 | 5.43517 | 21.60716 | 13.78357 |
|  | Ginsenoside Rg2 | 0.23032 | 0.21646 | 0.38380 | 0.01539 | 0.02418 | 0.00540 | 0.00420 | 0.01204 | 0.00273 | 0.30321 | 1.24304 | 1.51214 |

**Supplementary Table S4.** Splicing length distribution statistics table.

|  | min (bp) | max (bp) | median (bp) | mean (bp) | N50 (bp) | N90 (bp) |
| --- | --- | --- | --- | --- | --- | --- |
| Transcript | 176 | 16769 | 377 | 685 | 1078 | 273 |
| Unigene | 200 | 16769 | 286 | 490 | 590 | 231 |

**Supplementary Table S5.** Gene annotation success rate statistics table.

| Type | Number of Unigenes | Percentage (%) |
| --- | --- | --- |
| Annotated in NR | 269754 | 35.96 |
| Annotated in SwissProt | 110408 | 14.72 |
| Annotated in PFAM | 74099 | 9.88 |
| Annotated in GO | 45043 | 6 |
| Annotated in KO | 22879 | 3.05 |
| Annotated in all Databases | 3839 | 0.51 |
| Annotated in at least one Databases | 279109 | 37.2 |
| Totol Unigenes | 750245 | 100 |

**Table S6.** Expression profiles of 45 genes in the ginsenoside biosynthetic pathway.

| Pathway | Gene id | Average FPKM | | | | | | | | | | | |
| --- | --- | --- | --- | --- | --- | --- | --- | --- | --- | --- | --- | --- | --- |
|  |  | 1 year Rhizome | 2 years Rhizome | 3 years Rhizome | 1 year Stem | 2 years  Stem | 3 years Stem | 1 year  Lateralroot | 2 years Lateralroot | 3 years Lateralroot | 1 year Leaves | 2 years Leaves | 3 years Leaves |
| MVA pathway | ACAT1 | 154.10 | 88.89 | 58.45 | 76.28 | 184.22 | 7.13 | 36.36 | 160.63 | 96.31 | 379.40 | 366.87 | 98.29 |
|  | ACAT2 | 2337.29 | 5716.94 | 3683.51 | 223.69 | 692.55 | 142.10 | 597.90 | 2138.55 | 1601.35 | 1050.29 | 510.64 | 868.44 |
|  | ACAT3 | 562.57 | 455.11 | 482.89 | 139.72 | 430.11 | 43.21 | 141.31 | 356.04 | 218.36 | 759.60 | 663.13 | 336.37 |
|  | ACAT4 | 18.88 | 55.85 | 24.63 | 6.69 | 8.75 | 1.84 | 19.91 | 31.82 | 18.07 | 10.65 | 4.26 | 5.01 |
|  | ACAT5 | 185.04 | 427.84 | 352.65 | 21.31 | 55.56 | 13.72 | 35.18 | 167.10 | 120.96 | 108.13 | 74.80 | 76.70 |
|  | HMGS1 | 2068.00 | 2984.67 | 2797.00 | 183.33 | 549.33 | 75.00 | 301.00 | 978.67 | 730.33 | 966.33 | 395.33 | 679.33 |
|  | HMGR1 | 1123.33 | 4231.00 | 1847.00 | 12.00 | 16.33 | 1.33 | 44.00 | 481.33 | 131.33 | 107.67 | 27.00 | 5.33 |
|  | HMGR2 | 633.33 | 504.67 | 399.67 | 312.67 | 1388.67 | 80.33 | 114.67 | 311.67 | 178.00 | 343.00 | 253.67 | 231.00 |
|  | HMGR3 | 2068.00 | 2984.67 | 2797.00 | 183.33 | 549.33 | 75.00 | 301.00 | 978.67 | 730.33 | 966.33 | 395.33 | 679.33 |
|  | MVK1 | 366.16 | 1053.04 | 640.72 | 88.13 | 341.50 | 42.90 | 98.09 | 243.56 | 192.91 | 233.47 | 226.32 | 225.88 |
|  | MVK2 | 272.18 | 198.96 | 192.28 | 15.87 | 51.50 | 7.76 | 30.58 | 75.11 | 90.09 | 80.53 | 60.35 | 187.12 |
|  | PMK1 | 1015.33 | 859.33 | 681.00 | 305.67 | 711.00 | 68.00 | 130.67 | 463.33 | 362.33 | 611.67 | 604.00 | 889.00 |
|  | MVD1 | 3321.33 | 3172.67 | 2633.00 | 730.00 | 1529.67 | 280.67 | 695.33 | 1323.67 | 1015.00 | 1986.33 | 1256.67 | 1773.00 |
| MEP pathway | DXS1 | 1105.07 | 1243.91 | 903.20 | 309.62 | 513.73 | 13.67 | 156.27 | 639.50 | 528.41 | 207.79 | 1094.41 | 347.66 |
|  | DXS2 | 325.44 | 420.65 | 75.57 | 57.00 | 544.85 | 15.33 | 37.78 | 121.68 | 83.18 | 342.13 | 253.81 | 259.64 |
|  | DXS3 | 715.33 | 570.33 | 493.67 | 203.67 | 430.33 | 76.67 | 64.67 | 169.33 | 253.00 | 1294.00 | 961.00 | 1012.00 |
|  | DXS4 | 503.39 | 295.22 | 219.66 | 112.96 | 80.36 | 11.33 | 21.63 | 162.37 | 83.41 | 213.49 | 92.53 | 255.29 |
|  | DXR1 | 691.78 | 676.67 | 834.92 | 260.57 | 390.48 | 82.25 | 148.89 | 442.83 | 474.60 | 1116.03 | 724.67 | 1422.77 |
|  | DXR2 | 772.89 | 743.00 | 764.08 | 142.09 | 407.19 | 36.75 | 84.45 | 302.51 | 223.40 | 534.63 | 382.67 | 584.23 |
|  | MCT1 | 185.67 | 146.00 | 210.33 | 39.33 | 66.33 | 9.33 | 22.00 | 73.33 | 43.00 | 142.00 | 54.33 | 135.00 |
|  | CMK1 | 2017.67 | 2033.67 | 1952.67 | 228.00 | 372.00 | 87.00 | 395.33 | 581.67 | 516.00 | 1548.67 | 1233.00 | 1045.33 |
|  | MDS1 | 261.37 | 352.32 | 199.22 | 84.88 | 170.14 | 31.56 | 54.24 | 125.58 | 122.60 | 629.11 | 538.49 | 912.07 |
|  | MDS2 | 437.45 | 800.68 | 540.44 | 188.78 | 1153.23 | 97.11 | 124.08 | 530.59 | 473.34 | 1158.08 | 1288.84 | 2596.69 |
|  | HDS1 | 191.46 | 443.14 | 364.42 | 354.28 | 121.11 | 106.79 | 18.29 | 95.21 | 122.90 | 67.82 | 113.77 | 166.70 |
|  | HDS2 | 5980.98 | 4145.11 | 4784.81 | 1834.59 | 2989.16 | 590.33 | 728.02 | 1813.86 | 1772.51 | 11585.74 | 9968.89 | 10999.40 |
|  | HDS3 | 634.89 | 1108.76 | 4682.11 | 611.45 | 382.05 | 221.88 | 78.35 | 175.26 | 425.93 | 336.11 | 178.67 | 283.23 |
|  | HDS4 | 1074.06 | 1372.69 | 819.73 | 300.14 | 497.93 | 101.69 | 184.43 | 504.04 | 299.71 | 1787.97 | 1185.75 | 1796.23 |
|  | HDS5 | 1627.60 | 2351.64 | 1851.61 | 369.20 | 417.07 | 146.97 | 181.23 | 605.29 | 463.62 | 2396.70 | 973.59 | 2129.44 |
|  | HDR1 | 191.46 | 443.14 | 364.42 | 354.28 | 121.11 | 106.79 | 18.29 | 95.21 | 122.90 | 67.82 | 113.77 | 166.70 |
|  | HDR2 | 5980.98 | 4145.11 | 4784.81 | 1834.59 | 2989.16 | 590.33 | 728.02 | 1813.86 | 1772.51 | 11585.74 | 9968.89 | 10999.40 |
|  | HDR3 | 634.89 | 1108.76 | 4682.11 | 611.45 | 382.05 | 221.88 | 78.35 | 175.26 | 425.93 | 336.11 | 178.67 | 283.23 |
| Aglycone modifying enzyme | IDI1 | 467.17 | 375.97 | 171.12 | 109.56 | 188.63 | 13.17 | 116.73 | 273.25 | 163.37 | 310.18 | 292.56 | 349.83 |
|  | IDI2 | 2790.38 | 1984.30 | 1848.48 | 739.63 | 1623.92 | 178.83 | 614.93 | 1848.22 | 1352.18 | 1916.21 | 2092.11 | 2493.47 |
|  | IDI3 | 1142.45 | 1155.72 | 1393.06 | 386.14 | 1552.11 | 112.66 | 238.33 | 424.86 | 617.78 | 796.94 | 763.00 | 1100.71 |
|  | GPPS1 | 557.33 | 426.00 | 250.00 | 153.00 | 299.67 | 47.00 | 92.67 | 209.67 | 204.67 | 318.33 | 426.67 | 371.00 |
|  | FPPS1 | 543.33 | 1482.96 | 1088.52 | 43.31 | 531.19 | 44.78 | 288.86 | 945.63 | 733.48 | 245.52 | 171.97 | 335.75 |
|  | FPPS2 | 1746.00 | 4137.37 | 2437.81 | 118.02 | 433.48 | 81.89 | 432.48 | 1485.37 | 1113.52 | 525.81 | 321.69 | 596.25 |
|  | SQS1 | 853.75 | 1356.74 | 772.11 | 286.33 | 451.42 | 64.32 | 191.79 | 685.95 | 351.65 | 1414.46 | 855.72 | 774.33 |
|  | SQS2 | 702.15 | 1045.70 | 898.56 | 119.16 | 109.31 | 14.07 | 110.55 | 486.44 | 269.86 | 646.26 | 68.22 | 117.73 |
|  | SQS3 | 117.22 | 1094.40 | 739.59 | 539.88 | 538.71 | 113.95 | 166.56 | 535.78 | 565.68 | 864.58 | 1500.53 | 1031.74 |
|  | SQE1 | 415.00 | 280.67 | 258.00 | 109.33 | 179.33 | 45.67 | 47.67 | 126.00 | 136.33 | 222.00 | 273.33 | 316.33 |
|  | SQE2 | 776.52 | 232.00 | 195.84 | 47.67 | 97.23 | 25.10 | 46.55 | 85.00 | 93.55 | 137.77 | 159.22 | 304.73 |
|  | SQE3 | 263.97 | 266.17 | 267.44 | 63.37 | 463.10 | 66.86 | 56.19 | 158.90 | 144.94 | 279.50 | 482.20 | 392.41 |
|  | SQE4 | 638.72 | 465.88 | 416.22 | 86.20 | 173.60 | 41.85 | 69.21 | 168.56 | 141.62 | 286.93 | 309.32 | 330.83 |
|  | SQE5 | 197.37 | 264.50 | 207.89 | 55.30 | 961.57 | 81.14 | 59.47 | 259.44 | 188.39 | 626.17 | 1081.13 | 701.25 |

**Supplementary Table S7.** Pearson’s correlation between candidate genes and saponin content.

| Transcript_ID | Rg3 | F2 | Rd | Rb1 | Fa | Rc | Rb3 | Fc | Rg1 | R1 | Rg2 | Re | CE | ZR1 | IVa | Ro | RT1 | IV |
| --- | --- | --- | --- | --- | --- | --- | --- | --- | --- | --- | --- | --- | --- | --- | --- | --- | --- | --- |
| transcript/11063 | -0.041 | 0.053 | -0.035 | 0.010 | -0.018 | -0.279 | -0.284 | -0.256 | -0.231 | -0.260 | -0.011 | -0.354 | 0.575 | 0.470 | 0.548 | 0.466 | 0.456 | 0.468 |
| transcript/13633 | -0.089 | 0.203 | -0.029 | -0.360 | -0.046 | 0.416 | 0.448 | 0.241 | 0.381 | 0.082 | -0.175 | 0.239 | 0.369 | 0.271 | 0.373 | 0.288 | 0.274 | 0.287 |
| transcript/16431 | 0.249 | -0.006 | 0.219 | 0.260 | 0.239 | -0.334 | -0.335 | -0.295 | -0.072 | 0.145 | 0.500 | -0.119 | 0.773 | 0.750 | 0.701 | 0.627 | 0.634 | 0.639 |
| transcript/17023 | -0.549 | -0.480 | -0.534 | -0.454 | -0.463 | -0.262 | -0.225 | -0.178 | -0.242 | -0.429 | -0.302 | -0.454 | 0.753 | 0.740 | 0.807 | 0.832 | 0.839 | 0.837 |
| transcript/17465 | 0.042 | -0.098 | 0.067 | 0.118 | -0.027 | -0.113 | -0.108 | -0.141 | 0.113 | 0.194 | 0.275 | -0.029 | 0.802 | 0.755 | 0.748 | 0.659 | 0.651 | 0.660 |
| transcript/17513 | -0.019 | -0.183 | -0.027 | 0.034 | 0.009 | -0.266 | -0.256 | -0.214 | -0.072 | 0.012 | 0.241 | -0.195 | 0.874 | 0.809 | 0.806 | 0.713 | 0.725 | 0.730 |
| transcript/17578 | 0.025 | -0.164 | -0.022 | 0.110 | 0.165 | -0.351 | -0.355 | -0.110 | -0.183 | 0.000 | 0.240 | -0.230 | 0.457 | 0.397 | 0.394 | 0.308 | 0.316 | 0.315 |
| transcript/18868 | -0.342 | -0.366 | -0.379 | -0.297 | -0.136 | -0.302 | -0.275 | -0.030 | -0.216 | -0.233 | -0.103 | -0.340 | 0.474 | 0.440 | 0.505 | 0.488 | 0.479 | 0.481 |
| transcript/18959 | -0.184 | 0.052 | -0.182 | -0.312 | 0.051 | 0.022 | 0.032 | 0.161 | -0.022 | -0.200 | -0.231 | -0.146 | 0.313 | 0.178 | 0.292 | 0.187 | 0.184 | 0.193 |
| transcript/19015 | -0.624 | -0.470 | -0.620 | -0.553 | -0.501 | -0.262 | -0.222 | -0.145 | -0.298 | -0.515 | -0.415 | -0.509 | 0.600 | 0.575 | 0.664 | 0.688 | 0.696 | 0.690 |
| transcript/19333 | -0.155 | -0.207 | -0.167 | -0.119 | -0.116 | -0.347 | -0.329 | -0.288 | -0.171 | -0.141 | 0.129 | -0.345 | 0.894 | 0.855 | 0.878 | 0.825 | 0.827 | 0.830 |
| transcript/20333 | 0.287 | 0.090 | 0.244 | 0.300 | 0.212 | -0.411 | -0.418 | -0.367 | -0.149 | 0.136 | 0.533 | -0.173 | 0.503 | 0.550 | 0.468 | 0.456 | 0.454 | 0.456 |
| transcript/21526 | 0.183 | -0.050 | 0.119 | 0.255 | 0.175 | -0.451 | -0.456 | -0.360 | -0.295 | -0.031 | 0.374 | -0.272 | 0.352 | 0.336 | 0.307 | 0.264 | 0.273 | 0.265 |
| transcript/22148 | -0.195 | 0.065 | -0.139 | -0.396 | -0.161 | 0.357 | 0.391 | 0.203 | 0.300 | 0.005 | -0.241 | 0.146 | 0.400 | 0.289 | 0.387 | 0.292 | 0.292 | 0.298 |
| transcript/22438 | 0.511 | 0.104 | 0.471 | 0.622 | 0.365 | -0.409 | -0.437 | -0.428 | -0.141 | 0.251 | 0.707 | -0.068 | 0.480 | 0.507 | 0.376 | 0.328 | 0.346 | 0.346 |
| transcript/23326 | -0.288 | 0.023 | -0.218 | -0.402 | -0.324 | 0.119 | 0.150 | -0.020 | 0.140 | -0.105 | -0.196 | -0.109 | 0.695 | 0.631 | 0.647 | 0.576 | 0.603 | 0.607 |
| transcript/26023 | -0.602 | -0.480 | -0.606 | -0.534 | -0.493 | -0.290 | -0.252 | -0.173 | -0.320 | -0.506 | -0.373 | -0.511 | 0.552 | 0.564 | 0.621 | 0.671 | 0.683 | 0.676 |
| transcript/8255 | -0.291 | -0.139 | -0.301 | -0.309 | -0.278 | -0.288 | -0.262 | -0.288 | -0.258 | -0.330 | -0.118 | -0.433 | 0.631 | 0.566 | 0.621 | 0.571 | 0.583 | 0.583 |
| transcript/20381 | 0.118 | 0.179 | 0.234 | -0.015 | 0.074 | 0.877 | 0.884 | 0.685 | 0.831 | 0.574 | -0.108 | 0.845 | -0.292 | -0.294 | -0.278 | -0.290 | -0.317 | -0.303 |
| transcript/20527 | 0.364 | 0.254 | 0.458 | 0.303 | 0.232 | 0.731 | 0.718 | 0.523 | 0.761 | 0.692 | 0.159 | 0.852 | -0.342 | -0.316 | -0.339 | -0.343 | -0.379 | -0.364 |
| transcript/17372 | 0.857 | 0.622 | 0.854 | 0.731 | 0.806 | 0.086 | 0.037 | 0.017 | 0.304 | 0.553 | 0.720 | 0.449 | 0.039 | 0.048 | -0.008 | -0.061 | -0.095 | -0.077 |
| transcript/17415 | 0.727 | 0.456 | 0.752 | 0.767 | 0.635 | 0.040 | -0.013 | 0.040 | 0.331 | 0.625 | 0.702 | 0.400 | 0.149 | 0.170 | 0.086 | 0.029 | -0.005 | 0.011 |
| transcript/18520 | 0.875 | 0.501 | 0.819 | 0.750 | 0.792 | -0.155 | -0.192 | -0.202 | 0.133 | 0.534 | 0.903 | 0.315 | 0.092 | 0.160 | 0.051 | 0.034 | 0.010 | 0.018 |
| transcript/18740 | 0.702 | 0.418 | 0.699 | 0.638 | 0.652 | -0.022 | -0.058 | -0.057 | 0.258 | 0.516 | 0.717 | 0.327 | 0.363 | 0.360 | 0.304 | 0.233 | 0.205 | 0.221 |
| transcript/20743 | 0.804 | 0.482 | 0.746 | 0.811 | 0.654 | -0.244 | -0.298 | -0.331 | -0.118 | 0.269 | 0.672 | 0.129 | -0.223 | -0.187 | -0.256 | -0.258 | -0.283 | -0.274 |
| transcript/20914 | 0.851 | 0.537 | 0.785 | 0.611 | 0.832 | 0.003 | -0.020 | -0.056 | 0.167 | 0.476 | 0.760 | 0.429 | -0.248 | -0.175 | -0.269 | -0.262 | -0.279 | -0.273 |
| transcript/21226 | 0.731 | 0.517 | 0.737 | 0.648 | 0.706 | 0.023 | -0.019 | 0.026 | 0.309 | 0.563 | 0.709 | 0.370 | 0.238 | 0.245 | 0.183 | 0.117 | 0.085 | 0.102 |
| transcript/30028 | 0.524 | 0.486 | 0.540 | 0.442 | 0.550 | 0.013 | -0.026 | 0.010 | 0.103 | 0.181 | 0.356 | 0.159 | 0.156 | 0.103 | 0.070 | -0.011 | -0.008 | 0.006 |
| transcript/6636 | 0.624 | 0.551 | 0.651 | 0.424 | 0.609 | 0.291 | 0.269 | 0.229 | 0.464 | 0.589 | 0.514 | 0.538 | 0.008 | 0.009 | -0.062 | -0.131 | -0.145 | -0.129 |
| transcript/10310 | -0.058 | -0.153 | -0.047 | 0.242 | -0.110 | -0.381 | -0.410 | -0.249 | -0.308 | -0.191 | 0.024 | -0.353 | 0.139 | 0.181 | 0.166 | 0.217 | 0.208 | 0.203 |
| transcript/10660 | -0.308 | -0.305 | -0.310 | -0.133 | -0.263 | -0.294 | -0.284 | -0.146 | -0.275 | -0.281 | -0.148 | -0.395 | 0.372 | 0.302 | 0.321 | 0.264 | 0.287 | 0.283 |
| transcript/10735 | 0.109 | 0.308 | 0.129 | -0.050 | 0.273 | 0.159 | 0.148 | 0.196 | 0.113 | -0.033 | -0.081 | 0.065 | 0.164 | 0.028 | 0.127 | 0.012 | 0.000 | 0.015 |
| transcript/11520 | 0.127 | 0.239 | 0.168 | 0.126 | 0.196 | -0.066 | -0.085 | 0.014 | 0.072 | 0.043 | 0.119 | -0.061 | 0.479 | 0.414 | 0.455 | 0.389 | 0.367 | 0.384 |
| transcript/11558 | -0.053 | -0.014 | -0.095 | -0.103 | 0.195 | -0.221 | -0.237 | 0.001 | -0.209 | -0.209 | -0.021 | -0.264 | 0.258 | 0.203 | 0.247 | 0.200 | 0.207 | 0.205 |
| transcript/11879 | 0.217 | 0.107 | 0.216 | 0.371 | 0.245 | -0.375 | -0.410 | -0.216 | -0.219 | -0.050 | 0.275 | -0.247 | 0.355 | 0.354 | 0.307 | 0.286 | 0.287 | 0.295 |
| transcript/12573 | -0.140 | 0.033 | -0.146 | -0.238 | 0.183 | -0.022 | -0.021 | 0.261 | -0.003 | -0.116 | -0.149 | -0.115 | 0.267 | 0.159 | 0.245 | 0.153 | 0.146 | 0.157 |
| transcript/1277 | -0.380 | -0.124 | -0.352 | -0.463 | -0.226 | 0.076 | 0.105 | 0.115 | 0.020 | -0.249 | -0.351 | -0.174 | 0.461 | 0.339 | 0.468 | 0.386 | 0.386 | 0.388 |
| transcript/13211 | -0.306 | -0.027 | -0.282 | -0.288 | -0.256 | -0.122 | -0.112 | -0.087 | -0.170 | -0.326 | -0.303 | -0.338 | 0.314 | 0.233 | 0.334 | 0.297 | 0.287 | 0.292 |
| transcript/14336 | -0.102 | -0.252 | -0.109 | 0.113 | -0.085 | -0.485 | -0.491 | -0.339 | -0.303 | -0.184 | 0.156 | -0.434 | 0.752 | 0.736 | 0.732 | 0.706 | 0.708 | 0.709 |
| transcript/14408 | -0.092 | 0.057 | -0.045 | -0.178 | -0.106 | 0.062 | 0.079 | -0.039 | 0.112 | -0.034 | -0.036 | -0.058 | 0.702 | 0.609 | 0.630 | 0.525 | 0.544 | 0.553 |
| transcript/14507 | -0.416 | -0.430 | -0.432 | -0.320 | -0.368 | -0.394 | -0.360 | -0.306 | -0.300 | -0.326 | -0.099 | -0.492 | 0.849 | 0.799 | 0.839 | 0.800 | 0.815 | 0.814 |
| transcript/15190 | 0.144 | 0.051 | 0.097 | 0.202 | -0.028 | -0.517 | -0.516 | -0.587 | -0.361 | -0.130 | 0.339 | -0.386 | 0.404 | 0.449 | 0.423 | 0.453 | 0.442 | 0.440 |
| transcript/152 | -0.132 | -0.077 | -0.113 | -0.103 | -0.032 | -0.139 | -0.136 | -0.060 | -0.070 | -0.162 | -0.054 | -0.218 | 0.633 | 0.551 | 0.628 | 0.563 | 0.551 | 0.561 |
| transcript/15560 | 0.283 | 0.277 | 0.264 | 0.179 | 0.480 | -0.149 | -0.168 | -0.003 | -0.046 | -0.018 | 0.227 | -0.064 | 0.413 | 0.316 | 0.369 | 0.270 | 0.258 | 0.273 |
| transcript/16432 | -0.131 | -0.237 | -0.131 | -0.010 | -0.056 | -0.321 | -0.316 | -0.192 | -0.163 | -0.140 | 0.093 | -0.312 | 0.811 | 0.749 | 0.788 | 0.723 | 0.720 | 0.725 |
| transcript/16864 | -0.029 | 0.004 | -0.021 | 0.002 | -0.041 | -0.280 | -0.279 | -0.252 | -0.135 | -0.113 | 0.124 | -0.273 | 0.623 | 0.620 | 0.639 | 0.631 | 0.615 | 0.624 |
| transcript/17395 | -0.095 | 0.012 | -0.089 | -0.115 | 0.070 | -0.141 | -0.138 | 0.024 | -0.058 | -0.115 | -0.024 | -0.199 | 0.476 | 0.393 | 0.452 | 0.373 | 0.369 | 0.375 |
| transcript/17678 | 0.252 | 0.218 | 0.282 | 0.141 | 0.178 | -0.001 | 0.003 | -0.135 | 0.212 | 0.237 | 0.366 | 0.102 | 0.766 | 0.728 | 0.720 | 0.643 | 0.633 | 0.647 |
| transcript/17785 | 0.237 | 0.137 | 0.202 | 0.294 | 0.372 | -0.383 | -0.411 | -0.120 | -0.187 | 0.027 | 0.340 | -0.211 | 0.308 | 0.298 | 0.267 | 0.222 | 0.214 | 0.221 |
| transcript/17860 | -0.241 | -0.202 | -0.217 | -0.138 | -0.128 | -0.175 | -0.172 | -0.013 | -0.083 | -0.154 | -0.091 | -0.251 | 0.541 | 0.519 | 0.558 | 0.543 | 0.534 | 0.539 |
| transcript/18046 | 0.098 | 0.123 | 0.074 | 0.133 | 0.236 | -0.230 | -0.267 | -0.095 | -0.277 | -0.267 | -0.019 | -0.269 | 0.112 | 0.037 | 0.087 | 0.037 | 0.040 | 0.043 |
| transcript/20330 | -0.077 | 0.015 | -0.106 | -0.185 | 0.301 | -0.051 | -0.050 | 0.318 | -0.015 | -0.057 | -0.072 | -0.063 | 0.089 | 0.018 | 0.071 | 0.003 | -0.001 | 0.007 |
| transcript/20362 | -0.139 | -0.187 | -0.147 | -0.076 | -0.052 | -0.334 | -0.333 | -0.220 | -0.196 | -0.201 | 0.073 | -0.351 | 0.785 | 0.758 | 0.793 | 0.770 | 0.768 | 0.770 |
| transcript/20626 | -0.116 | -0.044 | -0.131 | -0.191 | 0.281 | -0.083 | -0.082 | 0.279 | -0.031 | -0.135 | -0.103 | -0.121 | 0.362 | 0.265 | 0.359 | 0.281 | 0.267 | 0.280 |
| transcript/21250 | 0.074 | 0.206 | 0.096 | 0.047 | 0.031 | -0.119 | -0.128 | -0.172 | -0.011 | -0.025 | 0.107 | -0.147 | 0.493 | 0.442 | 0.501 | 0.448 | 0.419 | 0.431 |
| transcript/22897 | -0.190 | -0.215 | -0.186 | -0.036 | -0.096 | -0.372 | -0.369 | -0.187 | -0.232 | -0.218 | 0.016 | -0.397 | 0.685 | 0.634 | 0.672 | 0.627 | 0.624 | 0.627 |
| transcript/22990 | -0.357 | -0.022 | -0.347 | -0.495 | -0.074 | 0.129 | 0.149 | 0.314 | 0.019 | -0.250 | -0.429 | -0.120 | 0.072 | -0.036 | 0.093 | 0.028 | 0.022 | 0.026 |
| transcript/23127 | -0.075 | -0.172 | -0.107 | -0.119 | 0.087 | -0.267 | -0.246 | -0.127 | -0.125 | -0.107 | 0.141 | -0.229 | 0.767 | 0.690 | 0.730 | 0.648 | 0.655 | 0.660 |
| transcript/24387 | 0.128 | 0.053 | 0.124 | 0.231 | 0.156 | -0.208 | -0.243 | -0.093 | -0.146 | -0.061 | 0.138 | -0.167 | 0.210 | 0.179 | 0.161 | 0.127 | 0.138 | 0.137 |
| transcript/24727 | -0.140 | 0.041 | -0.050 | -0.285 | -0.133 | 0.484 | 0.506 | 0.290 | 0.457 | 0.129 | -0.209 | 0.293 | 0.540 | 0.414 | 0.517 | 0.413 | 0.405 | 0.420 |
| transcript/25010 | -0.117 | -0.052 | -0.088 | -0.151 | -0.115 | -0.075 | -0.059 | -0.148 | 0.015 | -0.099 | 0.000 | -0.159 | 0.832 | 0.742 | 0.818 | 0.736 | 0.726 | 0.739 |
| transcript/25066 | 0.162 | 0.233 | 0.163 | 0.168 | 0.169 | -0.065 | -0.084 | 0.012 | 0.026 | 0.127 | 0.142 | -0.013 | -0.012 | -0.054 | -0.026 | -0.080 | -0.105 | -0.100 |
| transcript/26522 | -0.116 | -0.242 | -0.121 | -0.004 | -0.075 | -0.288 | -0.276 | -0.187 | -0.129 | -0.066 | 0.129 | -0.260 | 0.730 | 0.666 | 0.680 | 0.604 | 0.614 | 0.614 |
| transcript/2670 | 0.198 | 0.359 | 0.211 | 0.101 | 0.205 | -0.131 | -0.147 | -0.185 | -0.059 | -0.074 | 0.138 | -0.155 | 0.500 | 0.404 | 0.456 | 0.360 | 0.349 | 0.364 |
| transcript/27247 | -0.072 | -0.088 | -0.011 | -0.090 | -0.083 | 0.113 | 0.140 | 0.055 | 0.278 | 0.158 | 0.074 | 0.115 | 0.750 | 0.727 | 0.770 | 0.734 | 0.707 | 0.723 |
| transcript/27384 | -0.040 | -0.012 | -0.053 | -0.091 | 0.143 | -0.114 | -0.108 | 0.049 | -0.026 | -0.048 | 0.042 | -0.123 | 0.449 | 0.358 | 0.426 | 0.338 | 0.331 | 0.337 |
| transcript/2768 | 0.169 | 0.065 | 0.160 | 0.260 | 0.126 | -0.312 | -0.323 | -0.287 | -0.214 | -0.050 | 0.239 | -0.225 | 0.406 | 0.344 | 0.296 | 0.210 | 0.239 | 0.242 |
| transcript/28567 | 0.203 | 0.187 | 0.165 | 0.083 | 0.488 | -0.145 | -0.160 | 0.144 | -0.021 | 0.063 | 0.220 | -0.033 | 0.258 | 0.194 | 0.199 | 0.111 | 0.112 | 0.123 |
| transcript/30789 | -0.005 | 0.117 | 0.018 | -0.011 | 0.065 | -0.118 | -0.127 | -0.053 | -0.041 | -0.074 | 0.029 | -0.177 | 0.495 | 0.415 | 0.439 | 0.349 | 0.355 | 0.363 |
| transcript/32871 | 0.021 | 0.231 | 0.036 | -0.132 | 0.177 | -0.038 | -0.028 | 0.085 | 0.088 | 0.002 | 0.044 | -0.049 | 0.428 | 0.391 | 0.457 | 0.418 | 0.387 | 0.404 |
| transcript/35031 | 0.525 | 0.249 | 0.504 | 0.706 | 0.311 | -0.396 | -0.447 | -0.434 | -0.178 | 0.192 | 0.592 | -0.104 | 0.133 | 0.193 | 0.110 | 0.125 | 0.101 | 0.106 |
| transcript/3902 | 0.096 | -0.034 | 0.088 | 0.238 | 0.175 | -0.312 | -0.335 | -0.143 | -0.181 | -0.049 | 0.196 | -0.233 | 0.428 | 0.381 | 0.368 | 0.302 | 0.309 | 0.311 |
| transcript/4548 | 0.278 | 0.352 | 0.322 | 0.198 | 0.315 | 0.093 | 0.077 | 0.137 | 0.240 | 0.265 | 0.247 | 0.168 | 0.303 | 0.257 | 0.215 | 0.129 | 0.135 | 0.147 |
| transcript/5678 | 0.136 | 0.206 | 0.122 | 0.088 | 0.240 | -0.148 | -0.163 | 0.011 | -0.021 | 0.081 | 0.184 | -0.089 | 0.202 | 0.159 | 0.159 | 0.089 | 0.082 | 0.088 |
| transcript/5815 | -0.219 | -0.168 | -0.212 | -0.150 | -0.186 | -0.236 | -0.234 | -0.239 | -0.209 | -0.281 | -0.103 | -0.376 | 0.772 | 0.645 | 0.724 | 0.621 | 0.632 | 0.637 |
| transcript/5852 | 0.147 | 0.322 | 0.173 | -0.025 | 0.270 | 0.132 | 0.128 | 0.186 | 0.192 | 0.121 | 0.070 | 0.117 | 0.235 | 0.165 | 0.191 | 0.104 | 0.098 | 0.112 |
| transcript/6055 | 0.052 | 0.221 | 0.090 | -0.034 | 0.138 | -0.038 | -0.042 | -0.016 | 0.056 | -0.047 | 0.050 | -0.094 | 0.601 | 0.534 | 0.572 | 0.494 | 0.486 | 0.502 |
| transcript/6195 | -0.183 | -0.069 | -0.158 | -0.187 | -0.020 | -0.029 | -0.015 | 0.118 | 0.017 | -0.093 | -0.118 | -0.119 | 0.359 | 0.291 | 0.342 | 0.279 | 0.276 | 0.282 |
| transcript/6685 | -0.105 | -0.188 | -0.099 | -0.016 | -0.062 | -0.285 | -0.279 | -0.211 | -0.119 | -0.105 | 0.119 | -0.282 | 0.873 | 0.808 | 0.840 | 0.768 | 0.770 | 0.775 |
| transcript/6689 | 0.080 | 0.142 | 0.087 | -0.001 | 0.382 | 0.005 | -0.006 | 0.276 | 0.107 | 0.050 | 0.053 | 0.026 | 0.348 | 0.255 | 0.317 | 0.221 | 0.204 | 0.221 |
| transcript/701 | 0.383 | 0.605 | 0.386 | 0.149 | 0.424 | -0.030 | -0.053 | -0.106 | 0.022 | -0.001 | 0.212 | -0.015 | 0.288 | 0.212 | 0.258 | 0.177 | 0.158 | 0.178 |
| transcript/7442 | -0.161 | -0.245 | -0.153 | -0.079 | -0.081 | -0.250 | -0.237 | -0.146 | -0.096 | -0.114 | 0.070 | -0.260 | 0.857 | 0.796 | 0.832 | 0.765 | 0.766 | 0.771 |
| transcript/7643 | 0.090 | 0.119 | 0.072 | 0.078 | 0.336 | -0.214 | -0.231 | 0.053 | -0.128 | -0.137 | 0.056 | -0.168 | 0.238 | 0.202 | 0.286 | 0.270 | 0.229 | 0.244 |
| transcript/7674 | -0.192 | -0.214 | -0.181 | -0.054 | -0.193 | -0.342 | -0.336 | -0.279 | -0.223 | -0.231 | 0.012 | -0.386 | 0.708 | 0.689 | 0.722 | 0.710 | 0.705 | 0.707 |
| transcript/7959 | 0.158 | 0.105 | 0.124 | 0.089 | 0.457 | -0.088 | -0.095 | 0.260 | 0.038 | 0.133 | 0.182 | 0.053 | 0.022 | -0.002 | 0.007 | -0.041 | -0.056 | -0.049 |
| transcript/8058 | -0.156 | 0.289 | -0.062 | -0.282 | -0.176 | 0.363 | 0.368 | 0.176 | 0.230 | -0.132 | -0.390 | 0.057 | 0.229 | 0.088 | 0.223 | 0.135 | 0.120 | 0.136 |
| transcript/8314 | 0.398 | 0.474 | 0.378 | 0.262 | 0.307 | -0.229 | -0.250 | -0.336 | -0.104 | 0.031 | 0.371 | -0.119 | 0.299 | 0.300 | 0.293 | 0.266 | 0.242 | 0.256 |
| transcript/8619 | 0.145 | 0.048 | 0.103 | 0.109 | 0.312 | -0.377 | -0.374 | -0.137 | -0.198 | -0.051 | 0.310 | -0.222 | 0.415 | 0.425 | 0.365 | 0.337 | 0.356 | 0.358 |
| transcript/9550 | 0.049 | -0.008 | 0.038 | 0.162 | 0.102 | -0.396 | -0.416 | -0.266 | -0.297 | -0.236 | 0.127 | -0.353 | 0.404 | 0.418 | 0.426 | 0.450 | 0.445 | 0.445 |
| transcript/9665 | -0.155 | -0.191 | -0.154 | 0.049 | -0.214 | -0.407 | -0.421 | -0.367 | -0.327 | -0.264 | 0.017 | -0.471 | 0.643 | 0.576 | 0.588 | 0.531 | 0.544 | 0.546 |
| transcript/11920 | 0.309 | 0.254 | 0.281 | 0.297 | 0.387 | -0.246 | -0.269 | -0.131 | -0.152 | -0.017 | 0.272 | -0.114 | 0.096 | 0.080 | 0.084 | 0.058 | 0.044 | 0.049 |
| transcript/14144 | 0.569 | 0.367 | 0.526 | 0.538 | 0.495 | -0.321 | -0.357 | -0.337 | -0.150 | 0.111 | 0.556 | -0.036 | 0.114 | 0.191 | 0.143 | 0.188 | 0.156 | 0.166 |
| transcript/15054 | 0.529 | 0.511 | 0.548 | 0.654 | 0.351 | -0.166 | -0.230 | -0.208 | -0.036 | 0.219 | 0.402 | 0.009 | -0.162 | -0.144 | -0.206 | -0.223 | -0.246 | -0.238 |
| transcript/16907 | 0.698 | 0.558 | 0.626 | 0.535 | 0.863 | -0.169 | -0.216 | 0.024 | -0.029 | 0.246 | 0.556 | 0.158 | -0.312 | -0.274 | -0.315 | -0.318 | -0.348 | -0.338 |
| transcript/18518 | 0.622 | 0.536 | 0.559 | 0.477 | 0.633 | -0.188 | -0.226 | -0.118 | -0.053 | 0.244 | 0.536 | 0.111 | -0.357 | -0.267 | -0.338 | -0.296 | -0.327 | -0.319 |
| transcript/19002 | 0.429 | 0.371 | 0.360 | 0.309 | 0.583 | -0.269 | -0.292 | -0.083 | -0.174 | 0.027 | 0.362 | -0.067 | -0.098 | -0.107 | -0.113 | -0.143 | -0.159 | -0.152 |
| transcript/19495 | 0.327 | 0.295 | 0.259 | 0.268 | 0.527 | -0.274 | -0.305 | 0.029 | -0.173 | 0.059 | 0.290 | -0.079 | -0.299 | -0.269 | -0.301 | -0.298 | -0.317 | -0.311 |
| transcript/22225 | 0.641 | 0.487 | 0.569 | 0.535 | 0.570 | -0.248 | -0.284 | -0.222 | -0.069 | 0.318 | 0.639 | 0.083 | -0.236 | -0.172 | -0.264 | -0.266 | -0.284 | -0.281 |
| transcript/22260 | 0.637 | 0.505 | 0.622 | 0.682 | 0.478 | -0.250 | -0.301 | -0.313 | -0.123 | 0.166 | 0.524 | 0.005 | -0.118 | -0.075 | -0.153 | -0.148 | -0.163 | -0.156 |
| transcript/22811 | 0.656 | 0.412 | 0.591 | 0.610 | 0.578 | -0.355 | -0.393 | -0.353 | -0.185 | 0.163 | 0.650 | -0.018 | -0.062 | 0.023 | -0.074 | -0.040 | -0.052 | -0.050 |
| transcript/23637 | 0.648 | 0.687 | 0.703 | 0.622 | 0.431 | 0.127 | 0.074 | 0.031 | 0.372 | 0.599 | 0.563 | 0.381 | -0.213 | -0.098 | -0.213 | -0.179 | -0.222 | -0.210 |
| transcript/25701 | 0.718 | 0.430 | 0.655 | 0.647 | 0.627 | -0.244 | -0.279 | -0.248 | -0.035 | 0.346 | 0.719 | 0.151 | -0.109 | -0.011 | -0.113 | -0.079 | -0.099 | -0.097 |
| transcript/26781 | 0.375 | 0.357 | 0.358 | 0.387 | 0.158 | -0.094 | -0.130 | -0.295 | -0.121 | 0.042 | 0.212 | -0.020 | -0.176 | -0.215 | -0.194 | -0.223 | -0.245 | -0.238 |
| transcript/27310 | 0.733 | 0.484 | 0.686 | 0.686 | 0.782 | -0.100 | -0.153 | 0.038 | 0.072 | 0.411 | 0.621 | 0.274 | -0.357 | -0.306 | -0.377 | -0.381 | -0.416 | -0.405 |
| transcript/27547 | 0.648 | 0.444 | 0.579 | 0.455 | 0.793 | -0.135 | -0.163 | 0.003 | 0.053 | 0.311 | 0.607 | 0.195 | 0.044 | 0.033 | 0.009 | -0.049 | -0.072 | -0.060 |
| transcript/29163 | 0.576 | 0.439 | 0.569 | 0.620 | 0.445 | -0.141 | -0.190 | -0.207 | 0.001 | 0.243 | 0.473 | 0.079 | 0.042 | 0.015 | 0.016 | -0.036 | -0.074 | -0.062 |
| transcript/33512 | 0.628 | 0.369 | 0.564 | 0.600 | 0.631 | -0.342 | -0.382 | -0.278 | -0.185 | 0.123 | 0.581 | -0.014 | -0.010 | 0.045 | -0.001 | 0.022 | -0.007 | 0.003 |
| transcript/33759 | 0.807 | 0.467 | 0.750 | 0.851 | 0.731 | -0.297 | -0.357 | -0.200 | -0.036 | 0.442 | 0.797 | 0.162 | -0.232 | -0.138 | -0.257 | -0.234 | -0.266 | -0.259 |
| transcript/33994 | 0.669 | 0.369 | 0.588 | 0.612 | 0.820 | -0.313 | -0.360 | -0.060 | -0.097 | 0.284 | 0.671 | 0.089 | -0.157 | -0.076 | -0.188 | -0.178 | -0.190 | -0.184 |
| transcript/34644 | 0.863 | 0.571 | 0.812 | 0.769 | 0.828 | -0.123 | -0.173 | -0.106 | 0.096 | 0.467 | 0.772 | 0.299 | -0.165 | -0.122 | -0.190 | -0.209 | -0.246 | -0.235 |
| transcript/3481 | 0.875 | 0.542 | 0.806 | 0.802 | 0.836 | -0.260 | -0.313 | -0.204 | -0.016 | 0.421 | 0.838 | 0.201 | -0.159 | -0.075 | -0.195 | -0.187 | -0.209 | -0.201 |
| transcript/4965 | 0.459 | 0.308 | 0.446 | 0.635 | 0.241 | -0.369 | -0.414 | -0.348 | -0.154 | 0.225 | 0.527 | -0.095 | -0.146 | -0.031 | -0.155 | -0.101 | -0.119 | -0.121 |
| transcript/5283 | 0.563 | 0.472 | 0.536 | 0.421 | 0.711 | 0.009 | -0.028 | 0.183 | 0.180 | 0.359 | 0.460 | 0.292 | -0.259 | -0.187 | -0.209 | -0.175 | -0.226 | -0.214 |
| transcript/5546 | 0.636 | 0.435 | 0.557 | 0.317 | 0.811 | -0.045 | -0.054 | 0.007 | 0.073 | 0.224 | 0.546 | 0.262 | 0.004 | 0.026 | 0.023 | 0.013 | -0.013 | -0.002 |
| transcript/5912 | 0.299 | 0.341 | 0.266 | 0.213 | 0.379 | -0.205 | -0.226 | -0.086 | -0.087 | 0.073 | 0.287 | -0.085 | 0.102 | 0.068 | 0.067 | 0.007 | -0.008 | 0.002 |
| transcript/6503 | 0.361 | 0.437 | 0.354 | 0.194 | 0.455 | -0.016 | -0.039 | -0.036 | -0.007 | -0.008 | 0.181 | 0.021 | 0.230 | 0.118 | 0.163 | 0.056 | 0.056 | 0.070 |
| transcript/6759 | 0.761 | 0.527 | 0.725 | 0.733 | 0.578 | -0.251 | -0.302 | -0.352 | -0.021 | 0.354 | 0.741 | 0.112 | 0.022 | 0.101 | 0.016 | 0.038 | 0.005 | 0.013 |
| transcript/7174 | 0.472 | 0.325 | 0.403 | 0.303 | 0.792 | -0.146 | -0.172 | 0.179 | 0.007 | 0.178 | 0.413 | 0.129 | -0.076 | -0.064 | -0.058 | -0.069 | -0.103 | -0.090 |
| transcript/7398 | 0.747 | 0.523 | 0.666 | 0.523 | 0.687 | -0.223 | -0.248 | -0.293 | -0.037 | 0.308 | 0.747 | 0.148 | -0.040 | 0.037 | -0.063 | -0.055 | -0.059 | -0.061 |
| transcript/8399 | 0.541 | 0.394 | 0.523 | 0.666 | 0.308 | -0.308 | -0.364 | -0.331 | -0.075 | 0.320 | 0.594 | -0.026 | -0.085 | 0.008 | -0.100 | -0.073 | -0.103 | -0.100 |
| transcript/8713 | 0.641 | 0.508 | 0.588 | 0.563 | 0.730 | -0.175 | -0.227 | -0.003 | -0.017 | 0.292 | 0.536 | 0.147 | -0.367 | -0.291 | -0.364 | -0.336 | -0.369 | -0.358 |

**Table S8.** Pearson’s correlation between candidate proteins and saponin content.

| Protein_ID | Rg3 | F2 | Rd | Rb1 | Fa | Rc | Rb3 | Fc | Rg1 | R1 | Rg2 | Re | CE | ZR1 | IVa | Ro | RT1 | IV |
| --- | --- | --- | --- | --- | --- | --- | --- | --- | --- | --- | --- | --- | --- | --- | --- | --- | --- | --- |
| (brownCYP)transcript/13633 | -0.302 | -0.318 | -0.354 | -0.313 | -0.433 | -0.348 | -0.306 | -0.267 | -0.688 | -0.627 | -0.349 | -0.518 | -0.212 | -0.266 | -0.210 | -0.214 | -0.190 | -0.209 |
| (brownCYP)transcript/17465 | -0.360 | -0.368 | -0.395 | -0.367 | -0.434 | -0.219 | -0.172 | -0.137 | -0.508 | -0.555 | -0.388 | -0.472 | -0.101 | -0.143 | -0.091 | -0.095 | -0.079 | -0.092 |
| (brownCYP)transcript/18868 | -0.350 | -0.402 | -0.371 | -0.322 | -0.506 | -0.432 | -0.352 | -0.293 | -0.500 | -0.535 | -0.291 | -0.543 | 0.179 | 0.143 | 0.186 | 0.182 | 0.200 | 0.185 |
| (brownCYP)transcript/18959 | -0.414 | -0.305 | -0.455 | -0.489 | -0.226 | 0.416 | 0.375 | 0.341 | -0.297 | -0.468 | -0.612 | -0.228 | -0.591 | -0.643 | -0.574 | -0.582 | -0.570 | -0.578 |
| (brownCYP)transcript/19333 | -0.174 | -0.176 | -0.213 | -0.184 | -0.266 | -0.254 | -0.229 | -0.204 | -0.513 | -0.429 | -0.220 | -0.341 | -0.217 | -0.246 | -0.212 | -0.215 | -0.197 | -0.212 |
| (brownCYP)transcript/21526 | 0.089 | 0.018 | 0.020 | 0.092 | -0.206 | -0.668 | -0.685 | -0.669 | -0.871 | -0.425 | 0.059 | -0.408 | -0.277 | -0.272 | -0.269 | -0.267 | -0.249 | -0.271 |
| (brownCYP)transcript/22148 | -0.367 | -0.505 | -0.335 | -0.276 | -0.572 | -0.535 | -0.436 | -0.374 | -0.107 | -0.265 | -0.105 | -0.535 | 0.943 | 0.933 | 0.947 | 0.946 | 0.950 | 0.946 |
| (brownCYP)transcript/8255 | -0.161 | -0.174 | -0.215 | -0.172 | -0.315 | -0.398 | -0.359 | -0.318 | -0.697 | -0.546 | -0.222 | -0.422 | -0.303 | -0.348 | -0.304 | -0.308 | -0.284 | -0.303 |
| (purpleCYP)transcript/20381 | -0.283 | -0.142 | -0.275 | -0.351 | 0.067 | 0.807 | 0.752 | 0.696 | 0.340 | -0.007 | -0.449 | 0.176 | -0.400 | -0.423 | -0.386 | -0.391 | -0.399 | -0.390 |
| (yellowCYP)transcript/20914 | -0.253 | -0.093 | -0.256 | -0.335 | 0.087 | 0.753 | 0.765 | 0.752 | 0.292 | -0.065 | -0.451 | 0.207 | -0.560 | -0.572 | -0.538 | -0.542 | -0.551 | -0.544 |
| (brownUGT)transcript/11879 | -0.174 | -0.181 | -0.220 | -0.190 | -0.282 | -0.277 | -0.251 | -0.224 | -0.563 | -0.463 | -0.230 | -0.363 | -0.252 | -0.282 | -0.246 | -0.249 | -0.232 | -0.247 |
| (brownUGT)transcript/1277 | -0.463 | -0.330 | -0.483 | -0.530 | -0.219 | 0.493 | 0.534 | 0.547 | -0.074 | -0.423 | -0.644 | -0.139 | -0.518 | -0.567 | -0.499 | -0.506 | -0.500 | -0.503 |
| (brownUGT)transcript/152 | -0.095 | -0.144 | -0.163 | -0.095 | -0.350 | -0.616 | -0.597 | -0.560 | -0.902 | -0.590 | -0.136 | -0.520 | -0.284 | -0.307 | -0.277 | -0.278 | -0.253 | -0.278 |
| (brownUGT)transcript/17395 | 0.216 | 0.088 | 0.180 | 0.266 | -0.140 | -0.798 | -0.811 | -0.792 | -0.630 | -0.164 | 0.308 | -0.318 | 0.147 | 0.190 | 0.156 | 0.162 | 0.167 | 0.153 |
| (brownUGT)transcript/17860 | -0.125 | -0.164 | -0.171 | -0.109 | -0.352 | -0.599 | -0.509 | -0.431 | -0.714 | -0.541 | -0.133 | -0.456 | -0.161 | -0.186 | -0.156 | -0.158 | -0.136 | -0.157 |
| (brownUGT)transcript/22990 | -0.594 | -0.528 | -0.631 | -0.634 | -0.513 | 0.103 | 0.160 | 0.193 | -0.453 | -0.705 | -0.708 | -0.497 | -0.324 | -0.385 | -0.303 | -0.311 | -0.294 | -0.307 |
| (brownUGT)transcript/24727 | -0.313 | -0.140 | -0.275 | -0.374 | 0.126 | 0.957 | 0.991 | 0.981 | 0.686 | 0.119 | -0.457 | 0.340 | -0.293 | -0.309 | -0.277 | -0.282 | -0.297 | -0.282 |
| (brownUGT)transcript/25010 | -0.109 | -0.158 | -0.153 | -0.095 | -0.325 | -0.546 | -0.493 | -0.443 | -0.661 | -0.461 | -0.105 | -0.436 | -0.097 | -0.093 | -0.079 | -0.079 | -0.065 | -0.084 |
| (brownUGT)transcript/30789 | -0.066 | -0.038 | -0.106 | -0.096 | -0.098 | -0.087 | -0.080 | -0.068 | -0.392 | -0.302 | -0.166 | -0.160 | -0.397 | -0.418 | -0.393 | -0.395 | -0.384 | -0.394 |
| (brownUGT)transcript/6685 | -0.003 | -0.058 | -0.058 | 0.005 | -0.249 | -0.585 | -0.566 | -0.531 | -0.745 | -0.433 | -0.023 | -0.399 | -0.208 | -0.208 | -0.198 | -0.197 | -0.182 | -0.201 |
| (brownUGT)transcript/7674 | 0.203 | 0.185 | 0.139 | 0.185 | 0.008 | -0.436 | -0.454 | -0.443 | -0.670 | -0.257 | 0.108 | -0.161 | -0.497 | -0.473 | -0.484 | -0.481 | -0.471 | -0.488 |
| (yellowUGT)transcript/22225 | 0.388 | 0.233 | 0.420 | 0.469 | 0.139 | -0.505 | -0.536 | -0.546 | 0.156 | 0.432 | 0.620 | 0.086 | 0.729 | 0.779 | 0.711 | 0.718 | 0.709 | 0.715 |
| (yellowUGT)transcript/22260 | -0.237 | -0.251 | -0.277 | -0.246 | -0.341 | -0.273 | -0.240 | -0.210 | -0.537 | -0.491 | -0.273 | -0.406 | -0.161 | -0.205 | -0.160 | -0.164 | -0.145 | -0.159 |
| (yellowUGT)transcript/23637 | -0.136 | -0.209 | -0.074 | -0.078 | -0.127 | 0.023 | 0.051 | 0.055 | 0.489 | 0.232 | 0.071 | -0.012 | 0.825 | 0.818 | 0.811 | 0.811 | 0.802 | 0.815 |
| (yellowUGT)transcript/26781 | -0.177 | -0.019 | -0.180 | -0.249 | 0.121 | 0.635 | 0.677 | 0.687 | 0.246 | -0.068 | -0.372 | 0.231 | -0.596 | -0.618 | -0.584 | -0.588 | -0.592 | -0.587 |
| (yellowUGT)transcript/29163 | 0.606 | 0.673 | 0.583 | 0.554 | 0.661 | 0.208 | 0.117 | 0.069 | 0.105 | 0.437 | 0.443 | 0.584 | -0.681 | -0.659 | -0.702 | -0.698 | -0.701 | -0.697 |


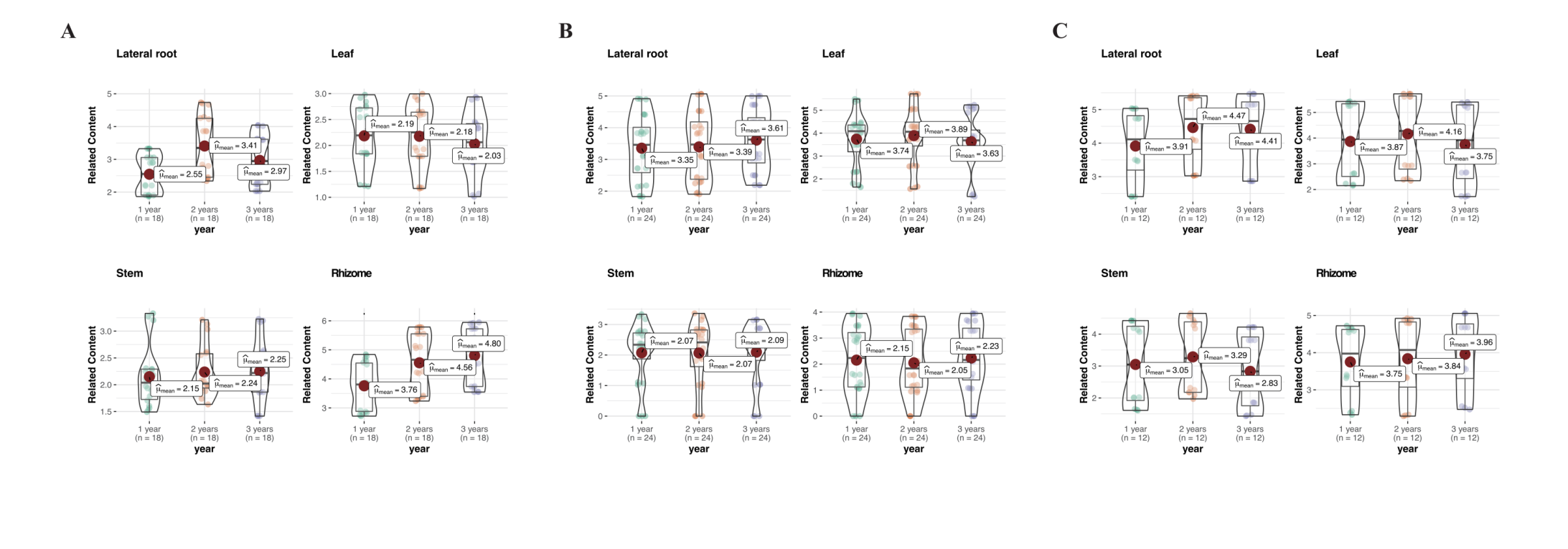


**Supplementary Figure S1.** Violin plots representing accumulation preference of three non-additively accumulated ginsenosides (A) OA type, (B) PPD type, and (C) PPT type in different tissues of *P. japonicus*. Each box extends from the lower to the upper quartile, and the line in each box represents the mean. The outer line extends to the minimum and maximum data points. The mean represents the accumulation of a certain type of ginsenoside in different tissues.


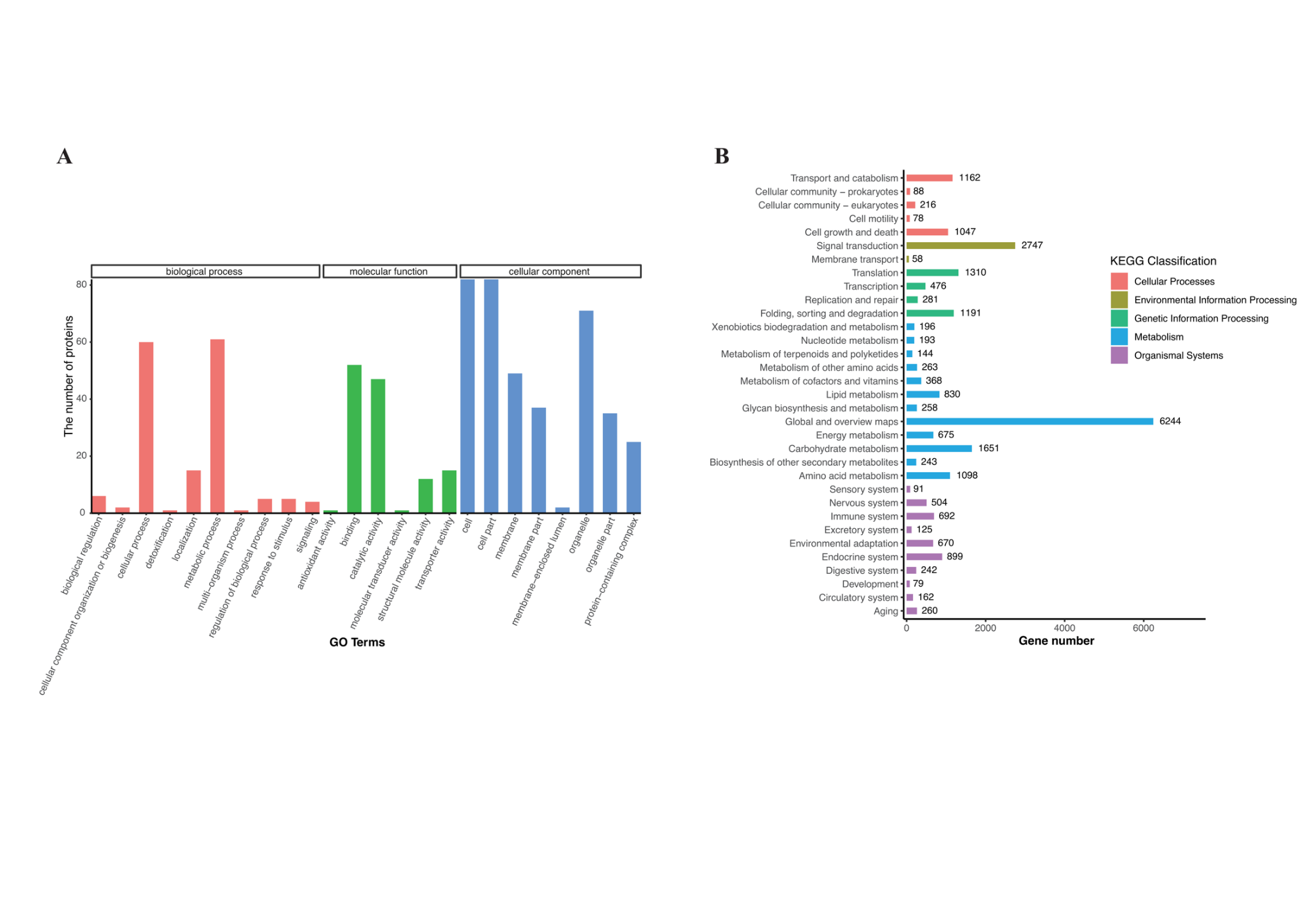


**Supplementary Figure S2.** Gene enrichment analysis. (A) GO enrichment analysis, (B) KEGG pathway enrichment analysis.

**
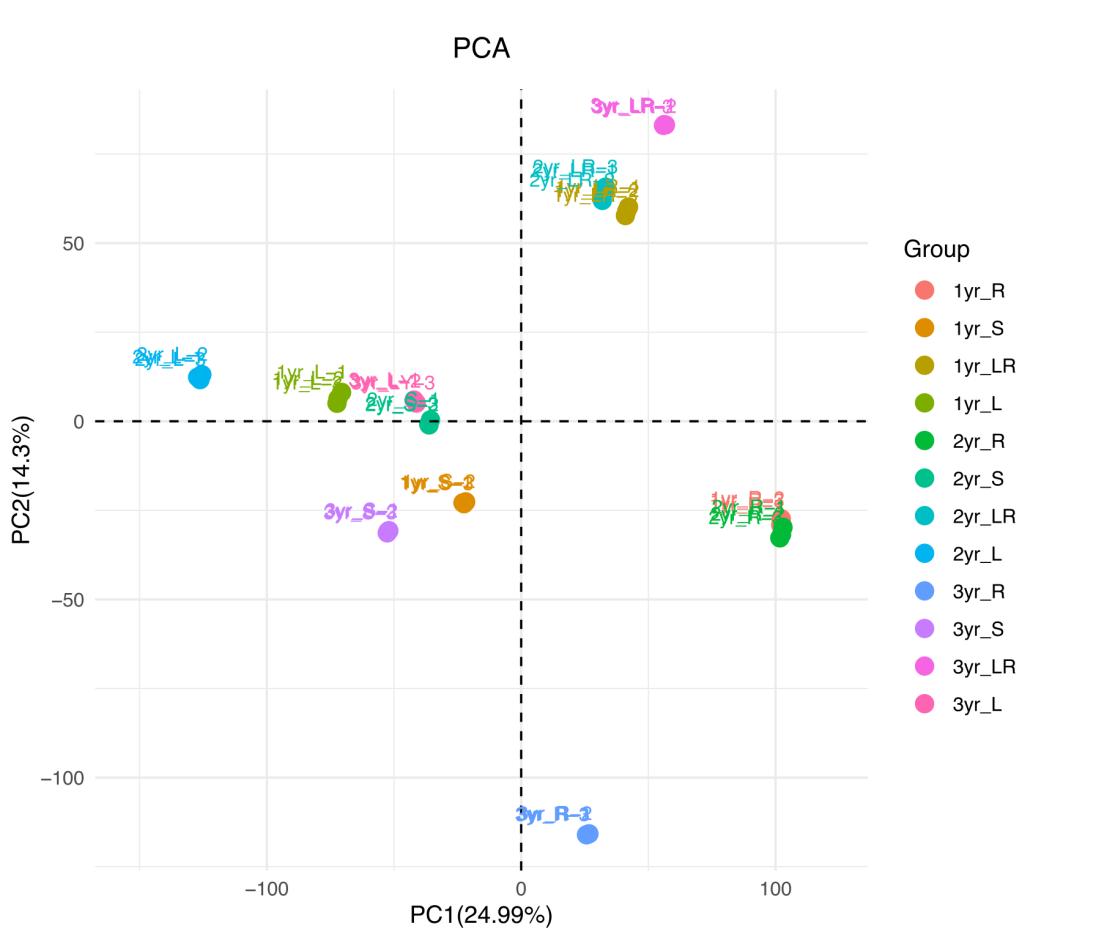
**

**Supplementary Figure S3.** The PCA results of the DEG groups between different samples. Principle compound analysis was carried out by the R ‘FactoMineR’ package.
